# The integrative analysis based on super-enhancer related genes for predicting different subtypes and prognosis of patient with lower-grade glioma

**DOI:** 10.3389/fgene.2023.1085584

**Published:** 2023-04-07

**Authors:** Yungang Hu, Qingqing Yang, Shuzhou Cai, Wei Wang, Shiyin Fu

**Affiliations:** ^1^ Department of Neurosurgery, Wuhan University of Science and Technology Affiliated Xiaogan Central Hospital, Xiaogan, Hubei, China; ^2^ Department of Thyroid and Breast Surgery, Wuhan University of Science and Technology Affiliated Xiaogan Central Hospital, Xiaogan, Hubei, China; ^3^ Department of Pediatric, Jinchu University of Technology Affiliated Central Hospital, Jingmen, Hubei, China

**Keywords:** super-enhancer, lower-grade glioma, prognostic signature, tumor immune microenvironment, immunotherapy

## Abstract

**Objective:** Emerging evidence revealed that super-enhancer plays a crucial role in the transcriptional reprogramming for many cancers. The purpose aimed to explored how the super-enhancer related genes affects the prognosis and tumor immune microenvironment (TIME) of patients with low-grade glioma (LGG).

**Methods:** In this study, the differentially expressed genes (DEGs) between LGG cohorts and normal brain tissue cohort were identified by the comprehensive analysis of the super-enhancer (SE) related genes. Then non-negative matrix factorization was performed to seek the optimal classification based on the DEGs, while investigating prognostic and clinical differences between different subtypes. Subsequently, a prognostic related signature (SERS) was constructed for the comprehensive evaluation in term of individualized prognosis, clinical characteristics, cancer markers, genomic alterations, and immune microenvironment of patients with LGG.

**Results:** Based on the expression profiles of 170 DEGs, we identified three SE subtypes, and the three subtypes showed significant differences in prognostic, clinicopathological features. Then, nine optimal SE-related genes were selected to construct the SERS through the least absolute shrinkage and selection operator Cox regression analysis. Survival analysis showed that SERS had strong and stable predictive ability for the prognosis of LGG patients in the The Cancer Genome Atlas, China Glioma Genome Atlas, and Remdrandt cohorts, respectively. We also found that SERS was highly correlated with clinicopathological features, tumor immune microenvironment, cancer hallmarks, and genomic alterations in LGG patients. In addition, the predictive power of SERS for immune checkpoint inhibitor treatment is also superior. The qRT-PCR results and immunohistochemical results also confirmed the difference in the expression of four key genes in normal cells and tumors, as well as in normal tissues and tumor tissues.

**Conclusion:** The SERS could be suitable to utilize individualized prognosis prediction and immunotherapy options for LGG patients in clinical application.

## 1 Introduction

Gliomas are the most common intracranial malignant tumor, accounting for more than 80% of primary malignant tumor in central nervous system (Ostrom et al., 2022). Low-grade gliomas (LGG), also known as WHO grade II and III tumors defined by the World Health Organization, are composed of diffuse low-grade and intermediate-grade gliomas (Brat et al., 2015). Compared with patients with glioblastoma (GBM), LGG patients have a relatively low degree of malignancy (Chen et al., 2022). However, even with comprehensive treatment including surgical resection, radiotherapy and chemotherapy, some LGG patients still have the characteristics of high recurrence and progression rates (Liu et al., 2018; Jiang et al., 2021). Significant heterogeneity in patient outcomes and treatment response remains a major clinical challenge for neurosurgeons. Traditionally, WHO grade II gliomas were considered to have a better prognosis than WHO grade III gliomas, but since the WHO reclassification of gliomas in 2016, molecular alterations have been considered more objective and precise than grading (Gittleman et al., 2020). Although there have been some progress in the onlooker research on LGG in recent years (Xu et al., 2021a), few drugs are currently approved for the treatment of LGG patients, and the prognosis has not been significantly improved (Ye et al., 2021). Therefore, there is an urgent need to explore new biomarkers to predict the prognosis of LGG patients and find potential therapeutic targets.

Gene regulation plays a major role in tumor pathogenesis, and the regulation of long non-coding RNAs (lncRNAs) on tumors is the hotspot of current research (Lou et al., 2020). Aberrant gene expression promotes tumorigenesis, progression and metastasis (Mansour et al., 2014). Enhancers in gene regulatory elements can bidirectionally transcribe enhancer RNA, a non-coding RNA transcribed by enhancers, that not only drives tumorigenesis, but also regulates genes and immune checkpoints (Lee et al., 2020). Super-enhancers (SE) are clusters of enhancers formed by contiguously arranged enhancers in tandem. SE usually appear near most of the key genes that determine cell identity and function, and play a more effective role than typical enhancers (Hnisz et al., 2013; Whyte et al., 2013). They have the ability to flexibly regulate, by combining unneeded regions to form highly concentrated regional transcriptional machinery, thereby affecting epigenetics and regulating tumorigenesis and progression (Chen et al., 2018). The researchers also found that SE operate covertly in a particularly latent manner, but control across multiple cancer lineages, with cancer cells assembling their own super-enhancers, thereby overproducing malignant oncogenes, exhibiting cancer hallmarks of hyperplasia, invasion and metastasis (Whyte et al., 2013). Yang believed that identifying, mapping out, and disrupting SE has the potential to transform how clinical cancer is managed (Whyte et al., 2013). Hence, as we concentrated on personalized therapy for patients with cancers, SE can serve as the potential biomarkers to track and understand the evolution of individual cancers, and ultimately may become important targets in therapeutic interventions.

The lncRNA HCCL5 in human tissue cells was identified as a SE-driven oncogenic factor that promotes the malignant development of hepatocellular carcinoma by promoting HCC cell viability, migration, and classical epithelial-mesenchymal transition (Peng et al., 2019). TCOF1 depletion in triple-negative breast cancer patients significantly inhibited the growth and invasiveness of triple-negative breast cancer cells (Hu et al., 2022a). Heparanase (HPSE) is a cancer metastasis protein that is regulated by the hnRNPU/p300/EGR1/HPSE axis, promotes high expression of HPSE enhancer RNA, is an independent prognostic factor for poor prognosis in cancer patients (Jiao et al., 2018). Regarding whether SE affect progression and overall survival in patients with LGG, the jury is still out.

Therefore, to solve the above problems, this study investigated the effect of different types of SE-related genes on the survival of patients with LGG by collecting data from The Cancer Genome Atlas (TCGA), China Glioma Genome Atlas (CGGA) and Rembrandt Database for LGG. At the same time, we constructed and evaluated prognostic score (SERS) based on 8 SE-related genes for patients with LGG. On top of that, the relationship was also explored between SERS and prognosis, clinicopathological features, tumor immune microenvironment, cancer hallmarks, genomic alterations and immunotherapy efficacy in patients with LGG. We provided a new strategy for predicting the prognosis of and assessing treatment effects for patients with LGG, and thus the findings of this study will help individualized prognosis prediction and immunotherapy decisions in patients with LGG.

## 2 Materials and methods

### 2.1 Data collection and study population

The RNA sequencing data and clinical information of LGG patients were extracted from TCGA (https://portal.gdc.cancer.gov/), CGGA (http://www.cgga.org.cn/) and Rembrandt (http://gliovis.bioinfo.cnio.es/) databases. A total of 5 LGG cohorts were gather in this study, namely, the TCGA, CGG693, CGGA325, CGGA301 and Rembrandt cohorts, respectively. Patients with no survival data or overall survival (OS) < 30 days were excluded from further analysis. Zakharova et al. (Zakharova et al., 2022) had re-classified the TCGA sampling according to the updated WHO CNS Tumor Classification in 2021, we therefore used the updated glioma diagnoses for analysis in this study. The transcriptome data with normal brain tissue were also obtained from Genotype-Tissue Expression (GTEx; https://gtexportal.org/home/). Furthermore, SE-related gene can be downloaded from the SEA v. 3.0 database (http://sea.edbc.org). The clinicopathological characteristics of LGG patients in five cohorts are generalized in Table 1.

**TABLE 1:** Characteristics of glioma patients in training and validation cohorts.

Clinicopathological characteristics	Training cohort	Validation cohorts			
	TCGA	CGGA693	CGGA325	CGGA325	Rembrandt
Number of patients	331	420	170	158	119
Age (mean ± SD; years)	41.3 ± 13.2	40.3 ± 10.4	40.4 ± 10.9	39.6 ± 10.6	NA
Gender					
Female	146	185	65	68	37
Male	185	235	105	90	59
NA	0	0	0	0	0
Survival status					
Alive	272	223	82	85	34
Dead	59	197	88	73	85
Preoperative KPS					
<80	50	NA	NA	NA	NA
≥80	93	NA	NA	NA	NA
NA	188	NA	NA	NA	NA
Histology					
Astrocytoma	193	254	110	102	80
Oligoastrocytoma	0	29	0	18	0
Oligodendroglioma	138	137	60	38	34
NA	0	0	0	0	0
WHO grade					
II	179	172	97	105	63
III	152	248	73	53	56
NA	0	0	0	0	0
IDH status					
Mutant	331	288	125	104	NA
Wild type	0	94	44	1	NA
NA	0	38	1	53	NA
1p19q codeletion					
Codeletion	138	125	55	16	8
Non-codeletion	193	257	113	33	13
NA	0	38	2	109	98
MGMT promoter status					NA
Methylated	271	200	84	43	NA
Unmethylated	60	129	70	106	NA
NA	0	38	16	9	NA
TERT status					NA
Mutant	116	NA	NA	NA	NA
Wild type	137	NA	NA	NA	NA
NA	78	NA	NA	NA	NA

The differential expression analysis firstly performed based on GTEx dataset and TCGA dataset, and finally 1,672 differentially expressed genes (DEGs) were extracted with the cutoff values of log2 fold-change |logFC|>2 and *p*-value < 0.05. Then, in the same way, 285 DEGs were extracted between GTEx dataset and TCGA dataset. Eventually, the differentially expressed SE-related genes were shared by two cohorts were considered eligible.

### 2.2 Identification of SE subtypes of LGG patients

Based on the above DEGs, non-negative matrix factorization (NMF) consensus clustering analysis was performed to obtain the optimal SE subtypes of LGG patients (Hillman et al., 2018). The commonality, dispersion and contour indicators are used to judge the optimal number of subtypes. The t-distributed stochastic neighbor embedding (tSNE) algorithm we applied to confirm the reliability of clustering results by naked eyes. The Kaplan-Meier survival curves were then used to identify differences in survival difference among different SE subtypes. In addition, we compared differences in clinicopathological features among different SE subtypes.

### 2.3 Construction and validation of a prognostic SERS

The univariate Cox regression was conducted to select the prognostic SE-related DEGs. Then the least absolute shrinkage and selection operator (LASSO) Cox regression analysis was performed to identify the SE-related prognostic signature (SERS) in the TCGA cohort (Friedman et al., 2010). The prognostic risk score of each LGG patient was calculated with the regression coefficient and the expression of the corresponding gene. The calculation formula of SERS was shown below:

$$Risk\;score=\sum_{i=1}^{n}\left({Coef}_{i}*X_{i}\right)$$

where n represents the number of all the selected gene; i represents the serial number of each gene; $X_{i}$ and ${Coef}_{i}$ refer to the expression level of each selected gene and corresponding coefficient, respectively. The cut-off value, defined as the median risk score was divide the patients into high- or low-risk group. The Kaplan-Meier survival curve analysis were conducted to evaluate the accuracy of prognosis of LGG patients between the high- and low-risk groups. The receiver operating characteristic (ROC) curves and the area under the ROC curves (AUC) were plotted and calculated to describe the accuracy of predicting OS. The above analyses were performed simultaneously in the TCGA cohort and four independent validation cohorts. What is more, we finally conducted meta-analysis to calculate the pooled hazard ratio of SERS.

### 2.4 Development of a nomogram

Initially, the univariate Cox regression analysis were performed based on SERS and clinicopathological features, and then multivariate Cox regression analysis was used to identify independent predictors in the TCGA cohort. The nomogram was developed in the TCGA cohort to individually predict 1, 3, and 5-year survival probabilities in LGG patients. And the predicted outcomes for LGG patients were presented in the form of ROC curves. To evaluate the stability of this nomogram, a 10-fold cross-validation algorithm was performed in the TCGA cohort for the internal validation, and the external validation was conducted in the other four independent cohorts. In addition, calibration curves and C-index were performed in the TCGA and validation cohorts to evaluate the usability of this nomogram.

### 2.5 Evaluation of genomic alterations

Tumor mutational burden (TMB) was calculated as the total number of somatic, coding, base substitution, and indel mutations examined per megabase of genome (Mayakonda et al., 2018). The somatic mutation profile ordered in Mutation Annotation Format (MAF) was obtained from the TCGA database. The mutation spectrum and frequency differences were analyzed between high and low risk genes (Bi et al., 2020). In addition, copy number alteration (CNA) data in LGG patients were obtained from the TCGA database. We used GISTIC2.0 to identify significant amplifications or deletions genome-wide. CNA burden was defined as the total number of genes with copy number changes at the focal and arm levels (Shen et al., 2019).

### 2.6 Assessment of TIME and immunotherapeutic responses

For the purpose of better understanding the underlying biological functions of DEGs between high-risk and low-risk groups, the Gene Ontology (GO) and Kyoto Encyclopedia of Genes and Genomes (KEGG) analyses were performed to identify annotated functions and gene enrichment pathways (Hu et al., 2022b). DEGs between high- and low-risk groups were set the cutoff values of |log2FC|>2 and the BH method adjusted *p* < 0.05.

There has been an increasing recognition that the interaction of cancer cells and tumor microenvironment may best be conceptualized as an ecological process (Kenny et al., 2006). Hence, the ESTIMATE algorithm was used to calculate the immune score, stromal score, ESTIMATE score and tumor purity in LGG patients (Yoshihara et al., 2013) for assessing the difference of stromal and immune cells in LGG. Simultaneously, CIBERSORT was performed to calculate the proportions of 22 immune cells from LGG based on gene expression (Newman et al., 2015). In addition, Tumor Immune Dysfunction and Exclusion (TIDE) algorithm was also applied to assess potential response to immune checkpoint inhibitions (ICI) therapy for LGG patients (Jiang et al., 2018).

### 2.7 Quantitative real-time polymerase chain reaction (qRT -PCR) and immunohistochemistry (IHC)

The normal human astrocyte line HA1800 and glioma cell lines U87, U251, A172 and LN229, were purchased from the Cell Bank of the Chinese Academy of Sciences. The clinical specimens of 10 LGG patients were collected in the Department of Neurosurgery of Wuhan Union Hospital from June 2021 to December 2021. Ten non-tumor brain tissues were obtained from patients with brain tissue resection due to craniocerebral injury from June 2021 to December 2021. The study was approved by the Medical Ethics Committee of our hospital, and the informed consent was obtained from each patient. Total RNA was extracted from cell lines and sample tissues using RNAiso Plus (Takara 9109). According to the instruction, cDNA was synthesized by reverse transcription through using HiScript^®^ III RT SuperMix for qPCR (+gDNA wiper) (Vazyme R323-01). The qRT-PCR analyses were performed using the AceQ^®^ qPCR SYBR Green Master Mix (Vazyme Q111-02) with PCR LightCycler480 (Roche Diagnostics, Basel, Switzerland). All expression data was normalized to GAPDH as an internal control using the 2^−ΔΔCT^ method. All primers used were synthesized by GeneCreate Biological Engineering Co., Ltd. (Wuhan, China). The protein levels of the selected genes were then verified by IHC experiments. In addition, the relations between the selected gene and tumor immune features also analyzed in LGG patients.

### 2.8 Statistical analysis

The PERL language (version, 5.30.2, http://www.perl.org) was used to preprocess RNA-seq transcriptome information. The R software (version 4.0.1, http://www.R-project.org) were conducted for statistical analyses and graph visualization. Continuous variables are described as mean ± standard deviation, and categorical variables are described as frequency (n) and proportion (%). Chi-square test or Fisher’s exact test was performed to compare categorical variables between two groups. Student’s *t*-test or one-way ANOVA was used to compare continuous variables with normal distribution between two or among more groups. The Mann-Whitney *U* test was used to compare non-normally distributed continuous variables between two groups, while the Kruskal Wallis test was used to compare non-normally distributed continuous variables among three or more groups. Survival differences between groups were assessed using Kaplan-Meier curves. Univariate and multivariate cox proportional hazards models were applied to estimate hazard ratios for variables and to identify independent prognostic factors. The cutoff value with statistical significance was set at two-tailed *p* < 0.05.

## 3 Results

### 3.1 Overall structure of this study

**FIGURE 1:**
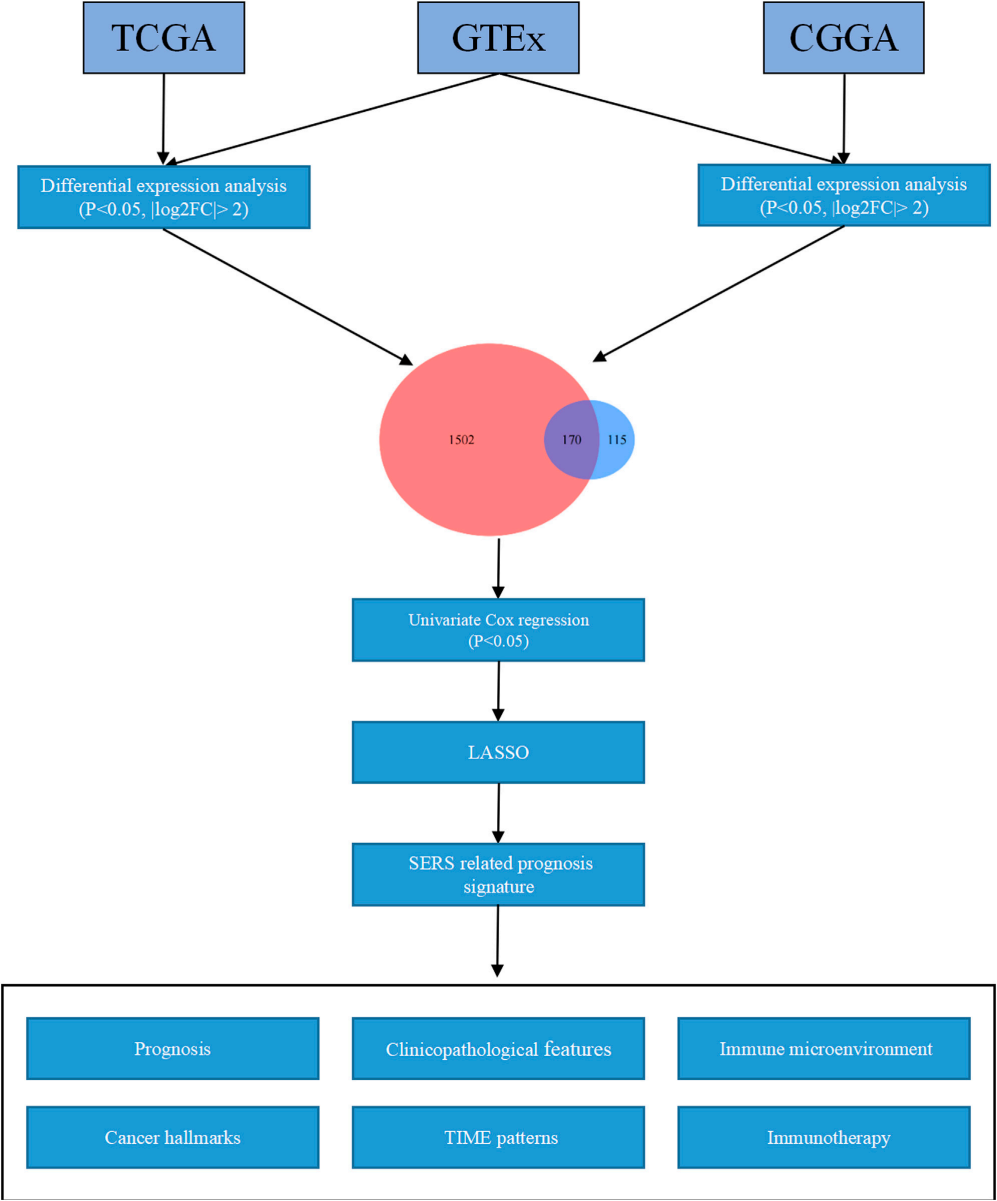
The overall flow chart of the study.

First of all, the GTEs between LGG and normal brain tissues were screen out. Based on the expression profiles of theses selected GTEs, NMF consensus clustering was performed to construct SE subtypes of LGG patients. Then, we explored the heterogeneities of prognosis and clinicopathological features for SE subtypes. Subsequently, the Univariate Cox regression analysis LASSO Cox algorithm were combined to screen for robust SERS and presented as a nomogram. The effectiveness of SERS was assessed in multiple dimensions. The overall flow diagram of this study was presented in Figure 1.

### 3.2 Identification of SE subtypes in TCGA cohort based on the DEGs

**FIGURE 2:**
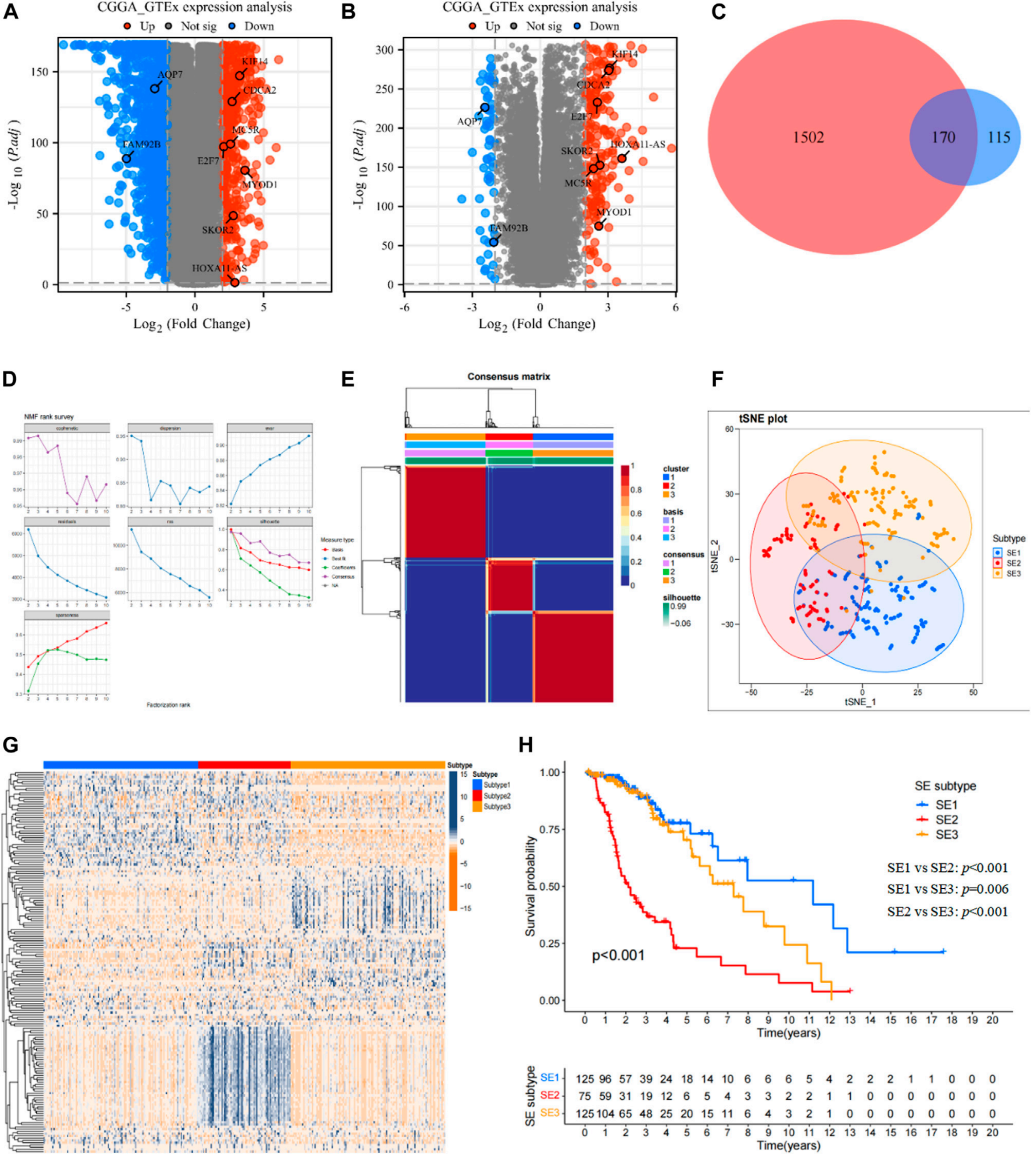
Identification of SE subtypes of LGG by using NMF algorithm. **(A)** Volcano plot showed DEGs (*p* < 0.05 and |log2FC|>2) between LGG tissues in TCGA cohort and normal brain tissues in GTEx database. **(B)** Volcano plot showed DEGs (*p* < 0.05 and |log2FC|>2) between LGG tissues in CGGA cohort and normal brain tissues in GTEx database. **(C)** Venn diagram identified prognostic super-enhancer related DEGs. **(D)** The NMF rank survey of TCGA cohort using theSE-related DEGs. **(E)** Consensus map of NMF clustering. **(F)** tSNE plot of 170 SE-related DEGs to distinguish SE subtypes. **(G)** Heatmap showed the expression levels of 170 SE-related DEGs among SE subtypes. **(H)** Kaplan–Meier survival analysis exhibited significantly different OS among three SE subtypes.

The differential expression analysis based on GTEx dataset and TCGA dataset was shown as the volcano in Figure 2A, and differential expression analysis based on GTEx dataset and CGGA693 dataset was also shown as the volcano in Figure 2B. Then, a total of 170 DEGs (Figure 2C) shared by two cohorts were used for subsequent analysis, they can be found in Supplementary Table S1.

Based on the expression profiles of 170 SE associated DEGs, the NMF was performed in the TCGA cohort to identify SE subtypes. As shown in Figure 2D, we chose 3 as the optimal number of clusters based on common, scatter, and contour metrics. Then, a total of 469 LGG patients were divided into three subtypes (Figure 2E), named SE1 (*n* = 125), SE2 (*n* = 75), and SE3 (*n* = 125). The heatmap of the consensus matrix exhibits clear boundaries, indicating the accuracy and robustness of the clustering results. t-SNE plot showed clear differences in the distribution between the three SE subtypes (Figure 2F). Significant differences in the expression of 170 prognostic SE-related DEGs can also be observed in the heatmap in Figure 2G. Kaplan-Meier survival curves showed obvious survival differences among the three SE subtypes (Figure 2H). The LGG patients in SE1 subtype had the best survival outcome, while SE2 had the worst survival outcome. At the same time, the heterogeneity of clinicopathological characteristics of these three subtypes were analyzed and found interestingly no significant differences among these clinicopathological characteristics (Supplementary Figure S1).

### 3.3 Development and validation of the SERS

**FIGURE 3:**
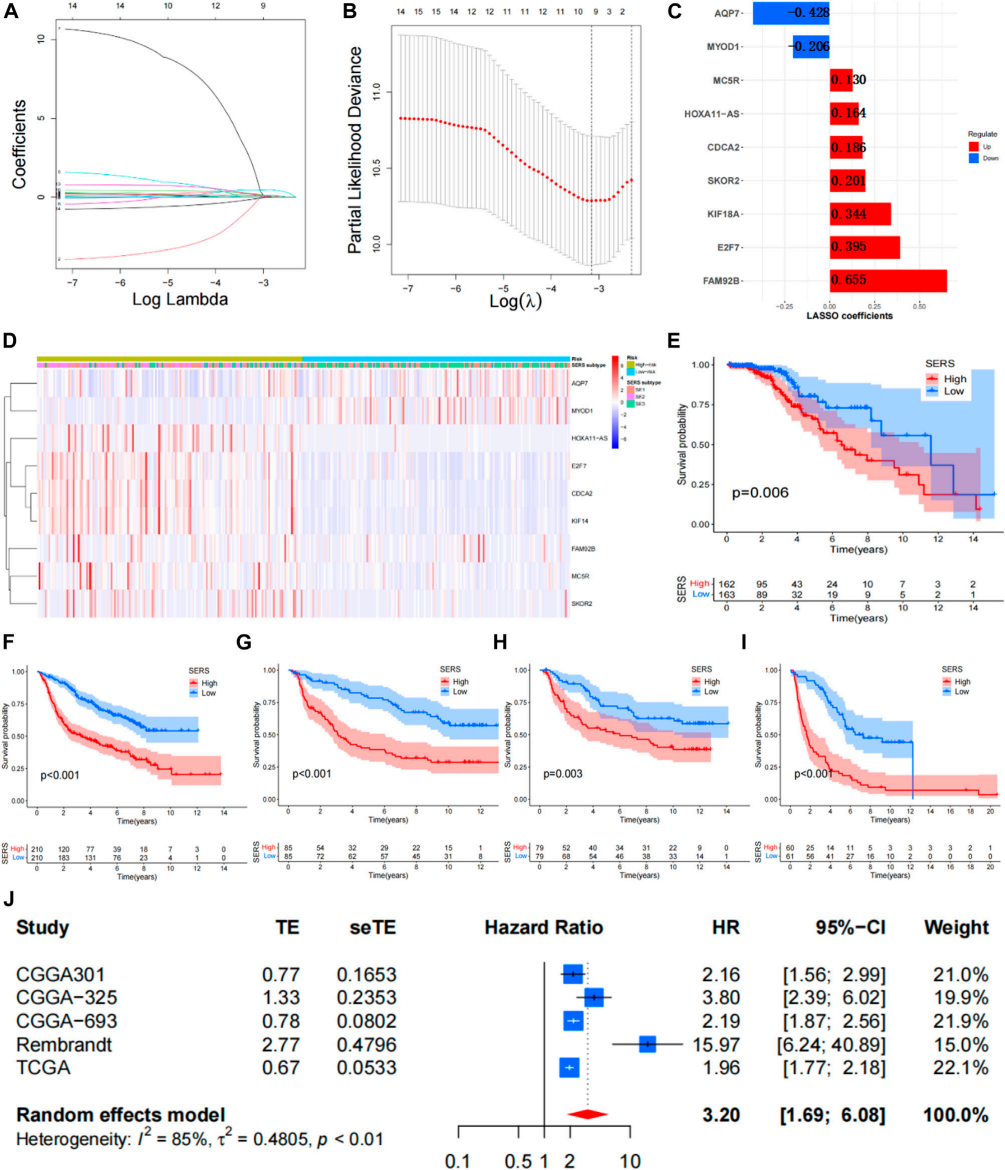
Construction of the SERS for LGG patients. **(A,B)** The LASSO regression was performed to minimize the risk of overfitting with the minimum criteria. **(C)** LASSO coefficients of selected the SERS. **(D)** Heatmap was represented expression levels of 8 SE-related genes in the high- and low-risk groups, respectively. **(E–I)** The Kaplan–Meier survival curves of SERS in TCGA, CGGA693, CGGA325, CGGA301, and Rembrandt cohorts, respectively. **(J)** Meta-analysis with random_effects showed a pooled hazard ratio (HR) of SERS.

The Univariate Cox regression analyses were conducted based on DEGs to identify prognostic SERG. The results of the analysis indicated 33 genes were obviously related to the OS of LGG, and detailed information for these prognostic SERG was shown in Supplementary Table S2. Then, the LASSO analysis was performed on above 33 prognostic SERG in the TCGA cohort to explore simplest and most accurate model. Finally, a total of 9 optimal prognostic SERG (AQP7, MYOD1, CDCA2, FAM92B, HOXA11-AS, E2F7, KIF18A, MC5R, and SKOR2) were stood out and incorporated in the SERS (Figures 3A, B). Figure 3C exhibited the LASSO coefficients of each selected gene in this signature. Among them, the coefficients of seven genes (CDCA2, FAM92B, HOXA11-AS, E2F7, KIF18A, MC5R, and SKOR2) are positive number, which means that they are related to bad prognosis for LGG patients, whereas the coefficients AQP7 and MYOD1 are negative number, indicating a good prognosis. The Kaplan-Meier survival curves of these nine optimal genes were shown in Supplementary Figure S2. The risk score of each patient was calculated as follows: SERS score = (−0.408 × AQP7) + (−0.107 × MYOD1) + 0.186 × CDCA2 + 0.625 × FAM92B + 0.163 × HOXA11 − AS + 0.454 × E2F7 + 0.344 × KIF18A + 0.130 × MC5R + 0.201 × SKOR2. Subsequently, the median SERS score in was set as the cut-off value to stratified the 325 LGG patients into the high- and low-risk groups. Heatmap analysis of nine genes showed markedly different distributions between high- and low-risk groups, the risky genes were upregulated in the high-risk group and the protective genes were upregulated in the low-risk group (Figure 3D).

The SERS was calculated with LASSO coefficients obtained from the TCGA cohort to stratified into with the median score high- and low-risk groups in other 4 cohorts. The Kaplan-Meier survival curves demonstrated that patients with high-SERS showed worse OS than low-SERS in the TCGA cohort (log-rank test *p* < 0.001; Figure 3E). Consistent results were also observed in four other independent validation cohorts (log-rank test *p* < 0.001; Figures 3F–I). The distribution plot of the risk score and survival status showed that the SERS had the positively correlation with the deaths of LGG patients (Supplementary Figures S3A–E). Furthermore, the ROC curves confirmed the satisfactory predictive performance of the of SERS in predicting 1-, 3-, and 5-year OS (Supplementary Figures S3F–I). Thus, SERS were sufficiently discriminative on both the validation cohorts. In addition, a meta-analysis was performed to assess the overall predicting accuracy, and the results indicated that the overall pooled HR for SERS was 3.2 (95% CI = 1.69–6.08; Figure 3J).

### 3.4 Relationship between SERS and clinicopathological characteristics, genomic alterations

**FIGURE 4:**
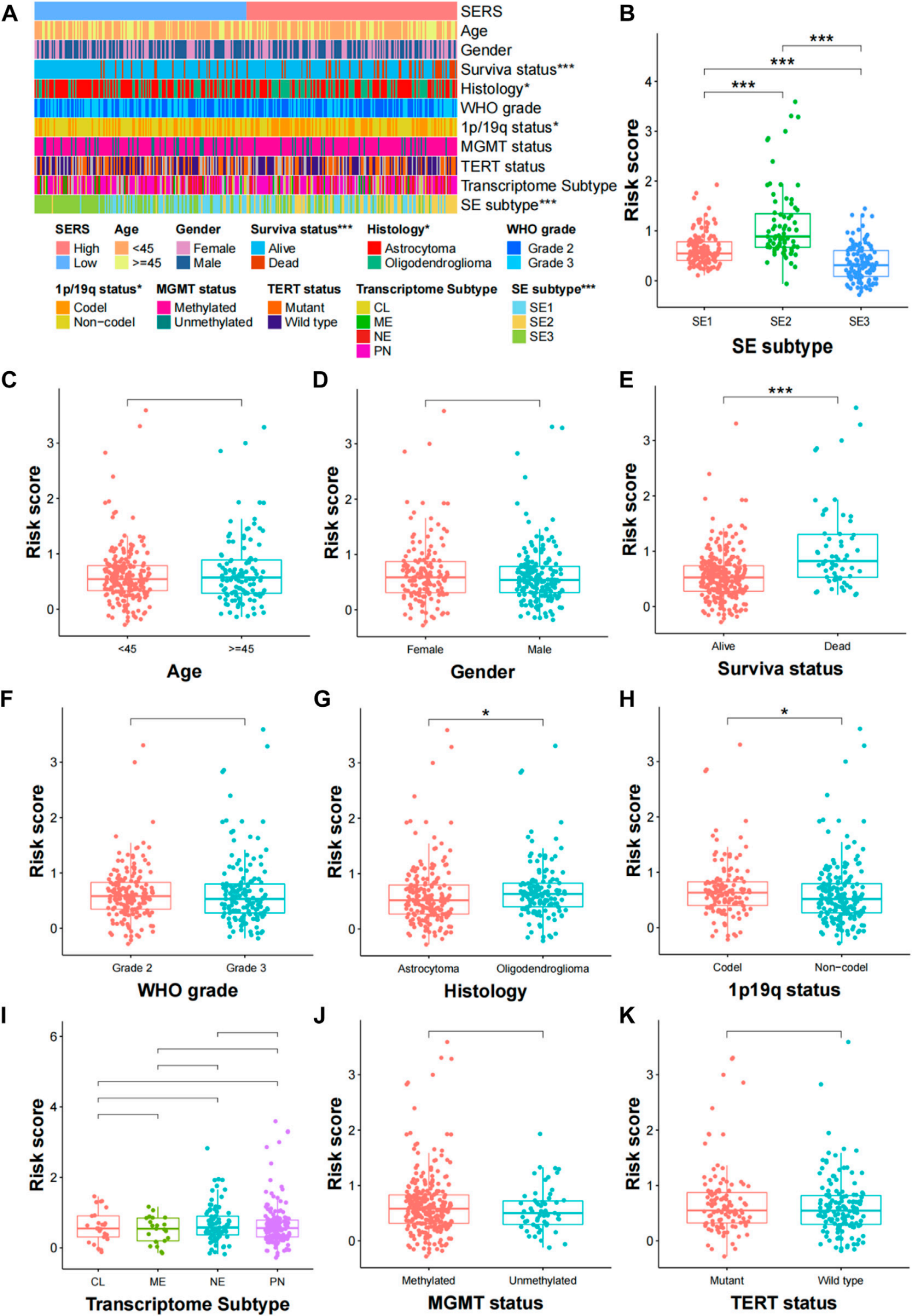
Correlation analysis between the prognostic SERS and clinicopathological characteristics in the TCGA cohort. **(A)** A heatmap was represented expression levels of eight selected SERS and the distribution of clinicopathological characteristics in the high- and low-risk groups, respectively. **(B–K)** Different levels of risk scores in glioma patients stratified by age, gender, Survival status, WHO grade, Histology, 1p19q codeletion, MGMT status, SERS subtype, TERT status and Transcriptome subtype. *p < 0.05, **p < 0.01, ***p < 0.001, and ns No significance.

The clinical relationships of SERS were attempted to explore in the TCGA cohort. As shown in Figure 4A, SERS were arranged from low to high to show the correlation between SERS and clinicopathological characteristics. There were significant differences in, survival status, Histology, 1p19q status, and SE subtype between high and low SERS groups, but no significant differences in -age, gender, WHO grade, MGMT status, TERT status and Transcriptome subtype. Furthermore, SERS levels between LGG patients stratified by various clinicopathological features were compared. The results of the analyses showed that LGG patients with, death status, Oligodendroglioma and SE2 subtype showed significant higher SERS, while no significant differences of SERS were observed in other subgroups (Figures 4B–K). Likewise, the relationship between SERS and clinicopathological characteristics of LGG patients in the CGGA693, CGGA325, CGGA301 and Rembrandt cohorts was also identified the similar results to the TCGA cohort (Supplementary Figures S4–S7).

**FIGURE 5:**
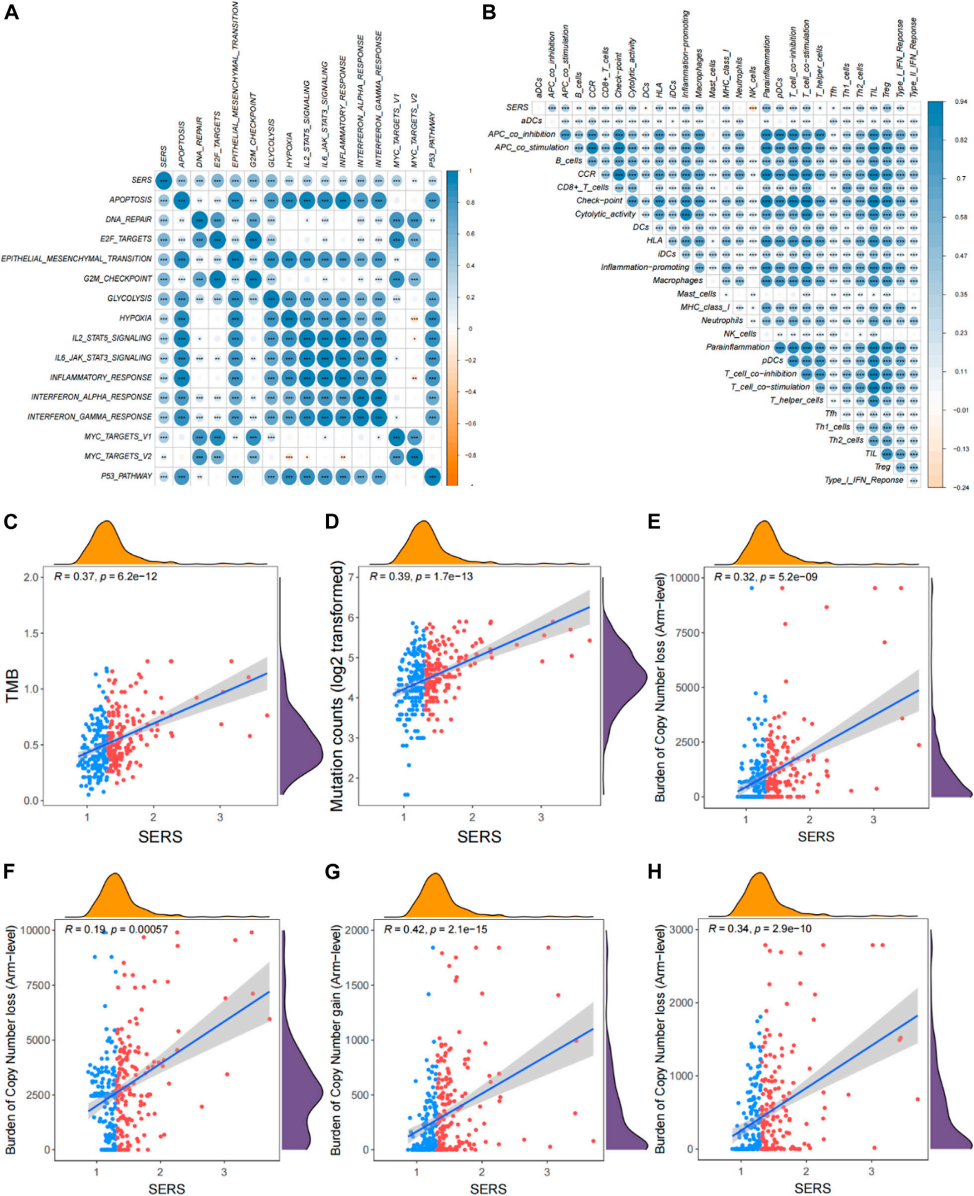
Correlation of SERS with clinicopathological features, genomic alterations and TIME patterns in TCGA cohort. **(A)** Correlation between SERS and the known cancer hallmarks of LGG patients. **(B)** Correlation between SERS and the 29 immune signatures. **(C–H)** Correlation of SERS with TMB, mutation counts, and copy number burdens at focal and arm levels. *p < 0.05, **p < 0.01, and ***p < 0.001.

To better address the prognostic features associated with SERS, the correlation between common cancer markers and SERS were also explored. The correlation heatmap showed that SERS was significantly positively correlated with many well-known cancer hallmarks including DNA repair, cell cycle, hypoxia, and metabolism (Figure 5A). The correlation between SERS and 29 immune signatures was illustrated by a correlation heatmap in TCGA cohort (Figure 5B). Subsequently, further analysis showed that SERS was significantly positively associated with TMB, mutation count, copy number gain and loss burden at the focal level, and copy number gain burden at the arm level (Figures 5C–H). The distribution of TMB, mutation counts, copy number burdens at focal and arm levels between high and low-risk groups were also compared in TCGA cohort (Supplementary Figures S10A–F). Based on the above data, it is indicated that high SERS may represent a higher frequency of genomic alterations to some extent.

### 3.5 Establishment and evaluation of a nomogram

**FIGURE 6:**
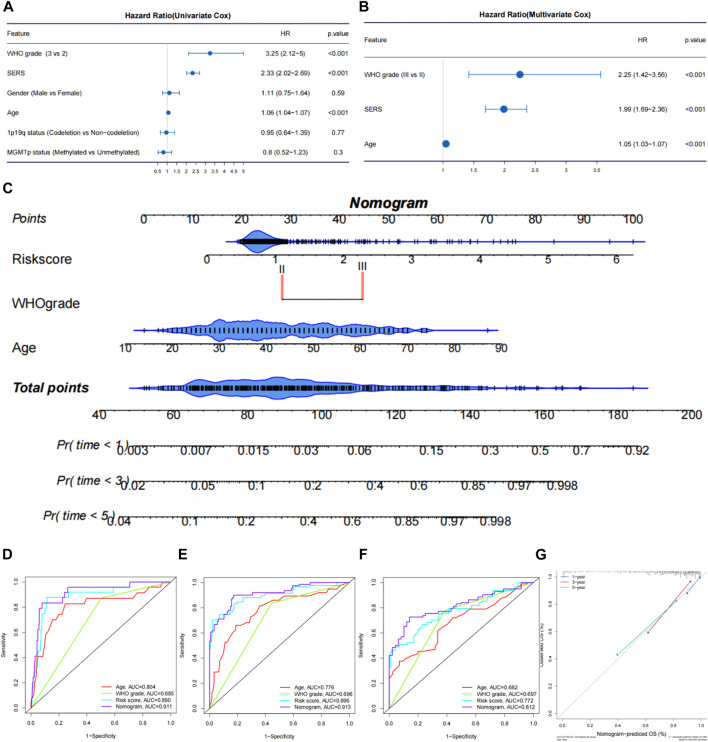
Establishment and evaluation of a nomogram in the TCGA cohort. **(A, B)** The univariate Cox regression and multivariate Cox regression were performed on SERS and other clinicopathological features TCGA cohort. **(C)** Nomogram based on SERS, WHO grade and age. **(D–F)** The receiver operating characteristic (ROC) curves of the nomogram predicted 1-, 3-, and 5-year OS in the TCGA cohort, respectively. **(G)** Calibration curves showed the good consistency between predicted and observed 1-, 3-, and 5-year overall survival (OS) in the TCGA cohort.

The univariate Cox regression and multivariate Cox regression analyses were performed to identify independent prognostic factors in the TCGA (Figures 6A, B), CGGA693, CGGA325, CGGA301, and Rembrandt cohorts (Supplementary Figures S8A–H). As we expected, SERS including nine selected genes was confirmed as an independent prognostic factor in all cohorts. The nomogram was established to predict 1-, 3-, and 5-year survival time in LGG patients based on the independent prognostic factors (age, WHO grade, and SERS) identified in the TCGA cohort (Figure 6C). The nomogram was firstly internally assessed, and the C-index was 0.862 (95% CI: 0.811–0.896), 0.833 (95% CI: 0.786–0.896), 0.812 (95% CI: 0.761–0.856) at 1, 3, and 5 years, respectively. The 1-year, 3-year and 5-year ROC curves showed that compared with SERS or age, the nomogram had the highest AUC values with the 1-year, 3-year and 5-year AUC values were 0.911, 0.913, and 0.812, respectively, which indicating that the nomogram had the optimal prediction effect (Figures 6D–F). The calibration curves showed a good fit between the actual and nomogram-predicted results for 1-, 3-, and 5-year OS (Figure 6G). In the same way, external validation of this nomogram was performed in the CGGA693, CGGA325, CGGA301 and Rembrandt cohorts. The accuracy in predicting 1-, 3-, and 5-year survival was good, and calibration curve analysis showed that the predicted and actual outcomes were basically conformity in all 4 cohorts (Supplementary Figures S9A–P). Therefore, this nomogram has potential as a quantitative predictor of prognosis in LGG patients.

### 3.6 Correlation of SERS with the LGG immune microenvironment and immunotherapy

**FIGURE 7:**
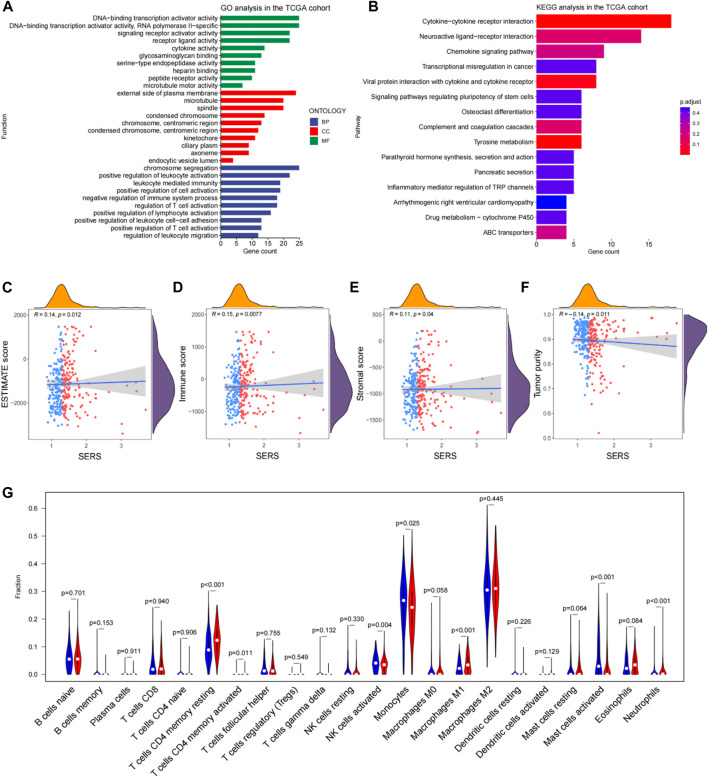
Functional enrichment analysis and immune landscape of glioma microenvironmental in the TCGA cohort. **(A, B)** Go analysis and KEGG analysis in the TCGA cohort. **(C–F)** Correlation of SERS with immune scores, stromal scores, ESTIMATE scores, tumor purity and SERS. **(G)** The abundance of 22 immune cells in the high- and low-risk groups. *p < 0.05, **p < 0.01, ***p < 0.001, and ns No significance.

Based on the differential expression analysis of high and low risk groups in the TCGA cohort, there were 462 DEGs (|log2FC|>2 and the BH method adjusted *p* < 0.05.) We then further performed functional enrichment analysis to characterize the biological functions of DEGs between the two risk subgroups. The results of GO analysis revealed that DEGs are enriched in several immune-related biological processes, such as regulation of T cell activation, positive regulation of T cell activation, negative regulation of immune system process, and positive regulation of lymphocyte activation (Figure 7A). Following, KEGG pathway analysis also showed significant enrichment of immune-related pathways, including cytokine-cytokine receptor interactions and chemokine signaling pathways (Figure 7B).

Given the findings that DEGs are enriched in immune-related functions, we further investigated the correlation of SERS with the immune microenvironment of LGG in the TCGA cohort. It turned out that SERS was significantly positively correlated with immune, stromal, and ESTIMATE scores, but negatively correlated with tumor purity, suggesting that the infiltration levels of immune cells and stromal cells increased with SERS (Figures 7C–F). The distribution of ESTIMATE scores, immune scores, stromal scores and tumor purity were no significant between high and low-risk groups in TCGA cohort (Supplementary Figures S10G–J). Further correlation analysis was performed between SERS and the infiltration levels of 22 immune cells quantified by the CIBERSORT algorithm. The results showed that the expressions of cells CD4 memory resting, T cells CD4 memory activated, NK cells activated, Monocytes, Macrophages M1, Mast cells activated and Neutrophils were significantly different in high and low risk groups. Among of them, the abundance of T cells CD4 memory resting, Macrophages M1, and Neutrophils was lower in the high-risk group, but the abundance of T cells CD4 memory activated, NK cells activated, Mast cells activated and Monocytes was higher in the high-risk group (Figure 7G).

**FIGURE 8:**
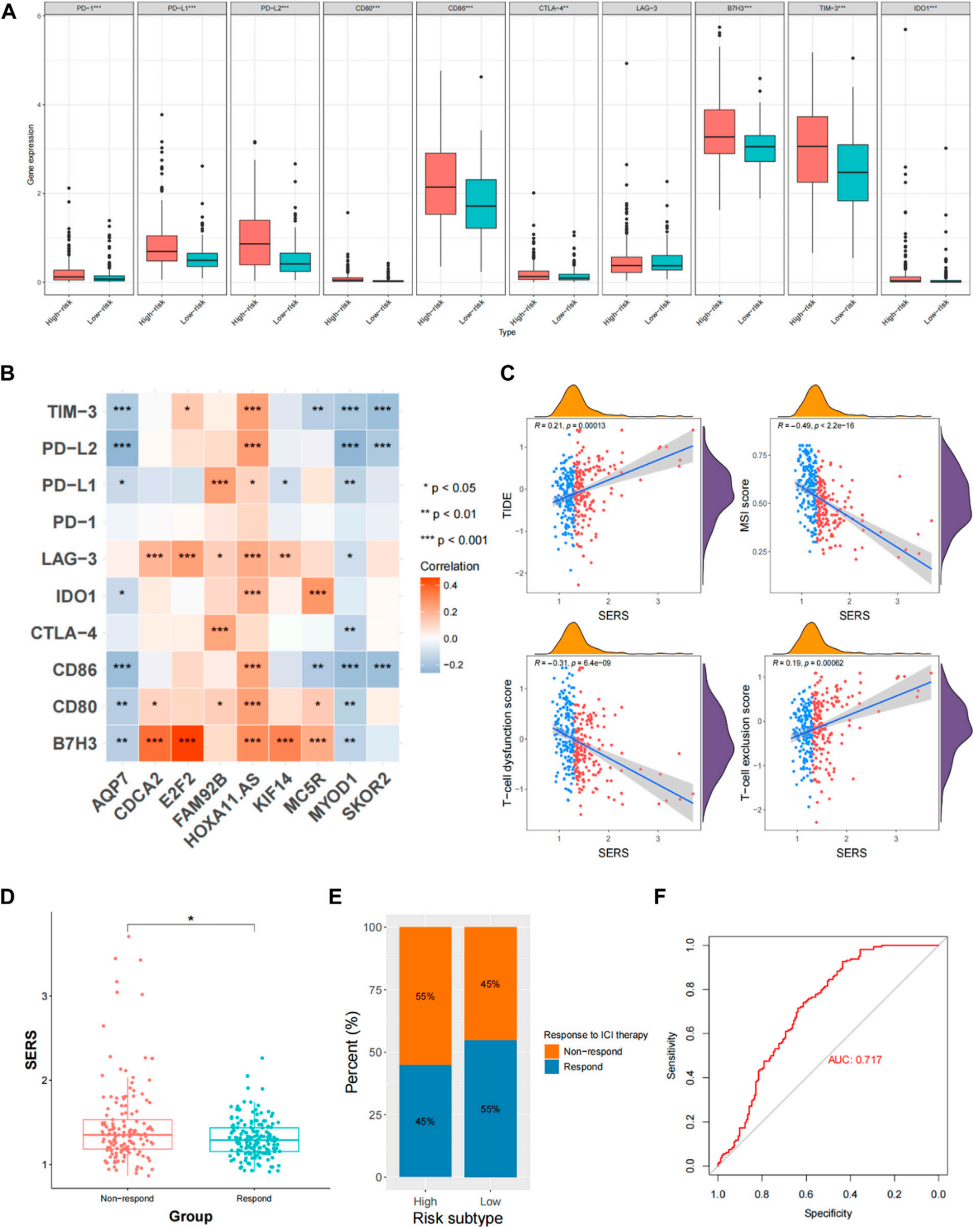
Evaluation of immune checkpoints and immunotherapy responsiveness in the TCGA cohort. **(A)** The expression levels of immune checkpoints and macrophage associated molecules in the high- and low-risk groups. **(B)** Correlation analysis between the prognostic SERS and immune checkpoints. **(C)** Correlation of SERS with T-cell dysfunction score, TIDE, MSI score and T-cell exclusion score. **(D)** The distributions of risk scores between non-respond and respond groups. **(E)** Comparative analysis of the response rates to ICI treatment in the high- and low-risk groups. **(F)** The ROC curve of predicting immunotherapeutic benefit.

In addition, we evaluated the correlation of SERS with immune checkpoints (PD-1, PD-L1, LAG-3, and B7-H3) and macrophage-associated molecules (CCL2, CCR2, CXCR4, and CSF1). The results showed that all immune checkpoints and macrophage-associated molecules were upregulated in the high-risk group except for LAG-3 (Figure 8A). We next determined whether there is a correlation between immune checkpoints and prognostic SERGs. The heat map showed that immune checkpoint proteins were significantly positively correlated with CDCA2, FAM92B, HOXA11-AS, E2F7, KIF18A, MC5R, and SKOR2, and significantly negatively correlated with AQP7 and MYOD1 (Figure 8B). In the TCGA cohort, SERS was positively correlated with TIDE and T-cell exclusion score, and negatively correlated with MSI score and T-cell dysfunction score (Figure 8C). The distribution difference can also be clearly observed in the high and low risk groups (Supplementary Figures S10K–N). In view of the TIDE algorithm, the distribution of SERS for the non-responder and responder groups to ICI indicated that the non-responder group had a significantly higher SERS, which just happened to explain the poorer prognosis of LGG patients who did not respond to ICI (Figure 8D). The high SERS subgroup had a lower proportion of responders to ICI treatment compared with the low SERS subgroup (*p* < 0.05, Figure 8E). Figure 8F showed that SERS had a satisfactory prediction in immunotherapy effect, which can provide a reference for whether patients should undergo immunotherapy.

### 3.7 The expression levels of selected SE-related genes

**FIGURE 9:**
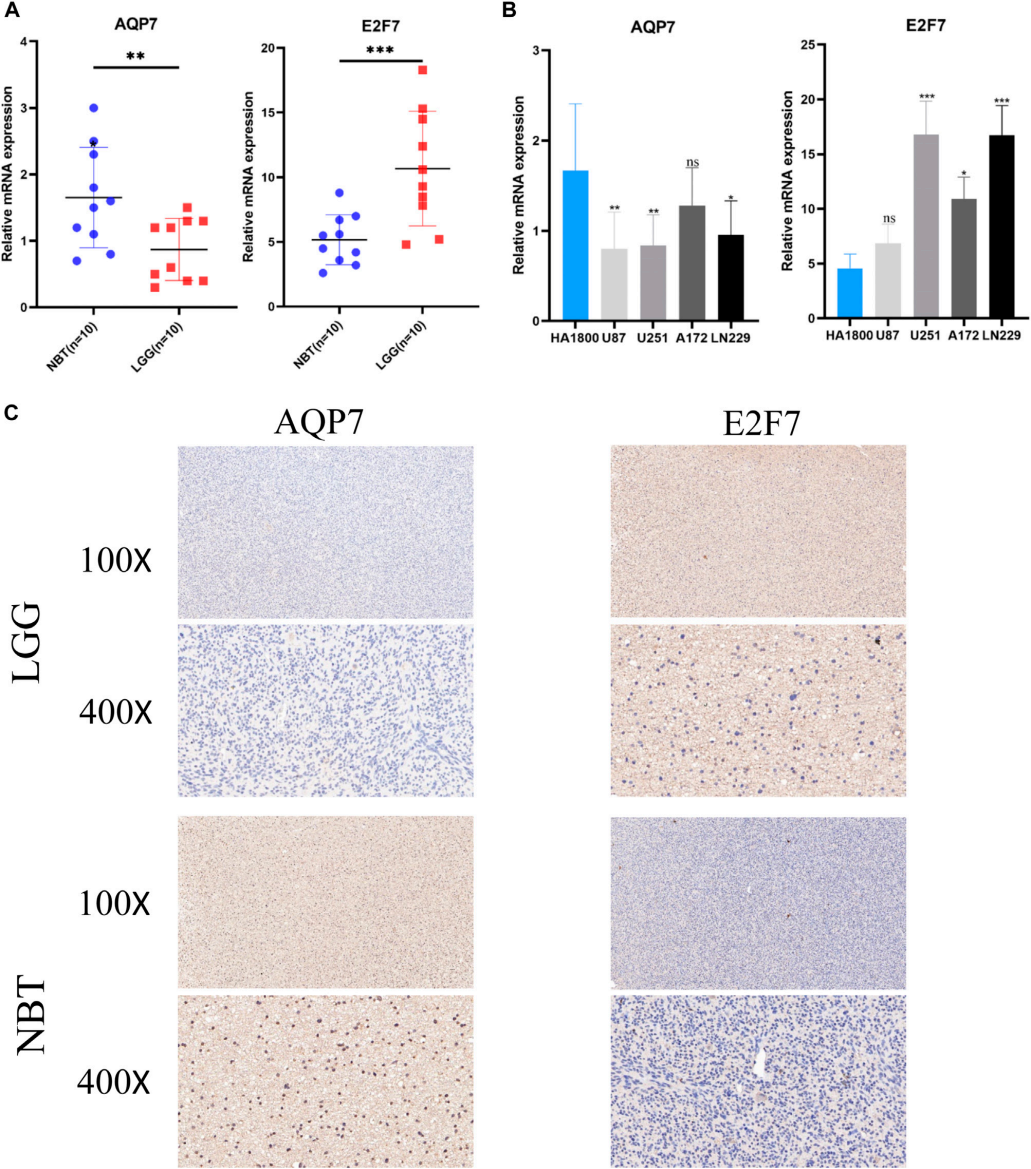
Validation of the expression levels of selected super-enhancer related genes. **(A)** Scatter plots of differential transcript levels between AQP7 and E2F7 in glioma cell lines and normal human astrocytes cell lines (HA1800). **(B)** Scatter plots of differential transcript levels between AQP7 and E2F7 in LGG and NBT. **(C)** The representative IHC staining images of AQP7 and E2F7. LGG low-grade glioma, NBT non-tumor tissues. *p < 0.05, **p < 0.01, ***p < 0.001, and ns No significance.

Two SE-related genes (AQP7, and E2F7) were selected to detect their transcriptional levels in cell lines, LGG tissues and normal brain tissues. The qRT-PCR results showed that compared with HA1800, the mRNA expression levels of AQP7 in human glioma cell lines were generally decreased, while the mRNA expression levels of E2F7 were generally increased (Figure 9A). Subsequently, we also detected their expression levels in 10 normal brain tissues and 10 glioma tissues. The qRT-PCR results of the tissue samples were consistent with those of the cell lines (Figure 9B). The representative IHC staining images of AQP7 and E2F7were shown in Figure 9C.

**FIGURE 10:**
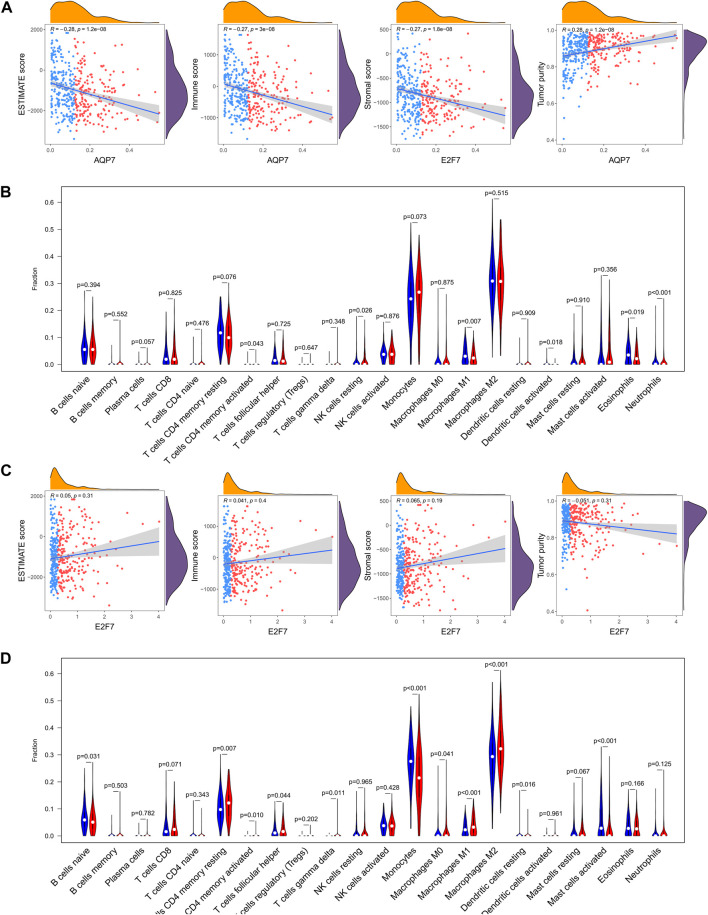
The relations between the selected gene and tumor immune features. **(A)** The abundance of 22 immune cells in the high-expression and low-expression of AQP7. **(B)** Correlation of the expression of AQP7 with immune scores, stromal scores, ESTIMATE scores, and tumor purity. **(C)** The abundance of 22 immune cells in the high-expression and low-expression of E2F7. **(D)** Correlation of the expression of E2F7 with immune scores, stromal scores, ESTIMATE scores, and tumor purity. *p < 0.05, **p < 0.01, ***p < 0.001, and ns No significance.

In addition, the relations between the selected gene (AQP7 and E2F7) and tumor immune features also analyzed. It turned out that AQP7 was significantly positively correlated with immune, stromal, and ESTIMATE scores, but negatively correlated with tumor purity (Figure 10A). While E2F7 was not significantly related with immune, stromal, and ESTIMATE scores, and tumor purity (Figure 10C). Further correlation analysis was also performed between the selected gene and the infiltration levels of 22 immune cells. The results showed that the expressions of 22 immune cells were significantly different whatever in high and low expression of AQP7 or E2F7 groups (Figures 10B, D).

## 4 Discussion

LGG patients, with the better prognosis than GBM patients, account for about half of all glioma patients. But their survival time varies widely, ranging from 1 year to more than 10 years (Chen et al., 2022), notwithstanding LGG patients with the same WHO grade had same standardized sequential therapy including surgery, radiotherapy, and chemotherapy. The high heterogeneity of LGG, which results in inconsistent treatment effects and prognosis, is a clinical conundrum faced by most neurosurgeons. And so, there is an urgent need to develop accurate and robust prognostic prediction models for data-assisted clinical decision-making. With the rapid development of bioinformatics and sequencing technologies, some studies have reported gene markers as prognostic indicators to predict the prognosis of LGG, such as hypoxia-related genes (Dao et al., 2018), ferroptosis-related genes (Wan et al., 2021), immune-related genes (Zhou et al., 2018) and the corresponding lncRNAs. Compared with other biomarkers, SE, as important distal regulatory DNA elements, are direct drivers of carcinogenesis and are highly tissue-specific. Therefore, SE are good candidates for predicting prognosis in various cancers. Ropri et al. (2021) and Huang et al. (2022) performed mechanistic exploration and prognostic prediction in breast cancer and hepatocellular carcinoma, respectively. However, whether super-enhancer related genes can serve as prognostic markers for LGG needs further discussion.

In this study, the NMF algorithm was used to identify three LGG subtypes in 325 LGG patients based on the expression profiles of DEGs between LGG and NBT. Then, significant differences in prognostic, clinicopathological features of the three LGG subtypes were observed with the naked eyes. A prognostic signature, called SERS, was constructed by univariate Cox regression and LASSO Cox regression for an individualized comprehensive assessment. The results showed that SERS was significantly associated with the prognosis, clinicopathological features, genomic alterations and TIME pattern of LGG patients, and the predictive ability of SERS for ICI treatment was also outstanding. In addition, a clinically accessible nomogram was constructed based on SERS, age, and WHO classification, which maintained excellent predictive accuracy in both the internal cohort and 4 external cohorts (CGGA693, CGGA325, CGGA301, and Rembrandt). So, it can provide a good net clinical benefit for screening LGG patients at high risk of death.

The SERS was constructed on 9 SE-related genes in our study, incorporating AQP7, MYOD1, CDCA2, FAM92B, HOXA11-AS, E2F7, KIF18A, MC5R, and SKOR2. Among these genes, CDCA2, FAM92B, HOXA11-AS, E2F7, KIF18A, MC5R, and SKOR2 were risky genes, which are associated with poor prognosis for LGG patients. Whereas the remaining two genes with good prognosis. Conversely, AQP7 and MYOD1 are related to good prognosis. AQP7, named Aquaporin 7, is a water and glycerol channel. Chen et al. demonstrated that low expression of AQP7 correlates with tumor grade and aggressive features of hepatocellular carcinoma (Chen et al., 2016). In a mouse model of breast cancer, lower AQP7 expression resulted in a reduction in primary tumor burden and lung metastases, thus suggesting that AQP7 is a prognostic indicator of overall survival in breast cancer patients (Dai et al., 2020). Myogenic differentiation 1 (MYOD1), as a transcription factor, promoted expression of muscle-specific genes. Wu et al. (2020) found that the expression of MYOD1D is positively correlated with the migration and invasion of gastric cancer cells. The cell division cycle associated 2 (CDCA2) has been proved to play an important role in the tumorigenesis of some cancers. The study showed that the high expression of CDCA2 was significantly correlated with the expression of related components of cell cycle phase transition and G2/M phase transition pathway, and suggested that CDCA2 could be a potential target for regulating tumor growth and radiation resistance in patients with oesophageal square cell carcinoma (Xu et al., 2021b). FAM92B, HOXA11-AS, E2F7, MC5R, and SKOR2 are important epigenetic regulators that can be targeted for cancer therapy. Specifically, E2F7 is an atypical E2F transcription factor family member with two independent DNA-binding domains. Some studies have found that E2F7 is upregulated in endometrial cancer, skin squamous cell carcinoma and other malignant tumors, promote tumor progression and metastasis in these cancers (Endo-Munoz et al., 2009; Li et al., 2015). KIF18A, a member of the kinesin-8 subfamily, has low expression in most human normal tissues and abnormally high expression in a variety of malignant tumor tissues (Marquis et al., 2021), which is associated with malignant pathological features and poor prognosis of cancer patients, and it promotes the proliferation, invasion and metastasis of tumor cells (Sepaniac et al., 2021). KIF18A may be a novel molecular targeted therapy for cancers. PTCRA (pre-T cell antigen receptor) is a protein-coding gene, together with the TCRB and CD3 complexes, encodes a protein that forms the T-cell pre-receptor complex, which regulates early T cell development (Liu et al., 2010).

The SERS is very effective in predicting prognosis of LGG patients in this study. To further explore the specific mechanism, we identified DEGs in high- and low-risk groups without hesitation. Then, the GO and KEGG analysis were performed to explore the detailed biological processes and pathways of these genes affecting the prognosis of LGG patients. Functional enrichment analysis revealed that DEGs between different risk subgroups were enriched in many immune-related biological processes and pathways. Therefore, we subsequently analyzed immune scores and immune cell infiltration between the two risk subgroups. Further analysis found that high risk was positively correlated with immune score, the abundance of T cells CD4 memory resting, and T cells CD4 memory activated. On the contrary, activated NK cells (tumor killer cells) showed higher abundance in the low-risk group. These results suggest that super-enhancer-related genes are related to the LGG immune microenvironment to a certain extent. From the above results, it can be concluded that the anti-tumor immunity of LGG patients in the high-risk group is significantly weakened, so we speculated that this may be one of the important reasons for their poor prognosis. The research of cancer immunotherapy has been very hot in recent years, especially immune checkpoint inhibitors have been quite mature as the first generation of immunotherapy, they play a therapeutic role in various cancers through mainly blocking PD-1/PD-L1 pathway and molecular receptors and/or ligands such as CTLA-4 (Topalian et al., 2015). Several previous studies have described therapeutic effect for immune checkpoints in some cancers, with findings consistent with favorable clinical outcomes in patients with many cancers, such as glioma (Puigdelloses et al., 2021), hepatocellular carcinoma (Sangro et al., 2020), lung cancer (Kartolo et al., 2021), and more. Therefore, we also evaluated the relationship between SERS and the expression levels of immune checkpoints, macrophage-related molecules, and immunotherapy response. It found that SERS was positively correlated with the expression levels of immune checkpoints and macrophage-related molecules. The response rate to ICI was significantly lower than that of the low-risk group. Therefore, we surmised that this may be another reason for the poor prognosis of LGG patients in the high-risk group. Taken together, the SERS proposed in our study can be used to screen clinically high-risk LGG patients, and then to prescribe professionally-informed treatment.

Without doubt, there are some inevitable shortcomings in this study. Firstly, this is a retrospective study based on public databases, thus yielding more reliable results in a prospective study. Secondly, the five cohorts of LGG patients have varying degrees of lack of clinical information, which may lead to varying degrees of selection bias. Thirdly, GO and KEGG enrichment analysis and subsequent immune microenvironment and immune checkpoint analysis were not validated in the other 4 cohorts. Fourthly, we only analyzed transcriptome information and did not perform multi-omics analysis including methylation and gene copy number. Finally, further experiments are needed to explore the specific molecular mechanism of super-enhancer related genes for further elucidation.

## 5 Conclusion

In conclusion, three novel LGG subtypes were established based on SE-related genes. Subsequently, an accurate and independently validated model were proposed for predicting overall survival in LGG. In addition, we also found that SERS was associated with prognosis, clinicopathological features, tumor immune microenvironment, cancer hallmarks, and genomic alterations and the effect of immunotherapy in patients with LGG. The findings can be as the novel biomarkers for predicting prognosis and potential therapeutic targets for LGG, which will help physicians and patients to evaluate prognosis, determine follow-up period, and make immunotherapy decisions.

## Data Availability

The original contributions presented in the study are included in the article/Supplementary Material, further inquiries can be directed to the corresponding author.

## Supplementary material

SUPPLEMENTARY FIGURE S1 Comparisons of age, gender, KPS, survival status, histology, WHO grade, MGMT status, Transcriptome subtype and TERT status among SE subtypes in TCGA cohord.

SUPPLEMENTARY FIGURE S2 The Kaplan–Meier curves of TCGA cohort showed that there is different overall survival for patients with different expression levels of nine selected super-enhancer related prognostic genes.

SUPPLEMENTARY FIGURE S3 Risk scores, survival status in high and low-risk group and Kaplan–Meier survival curves of SERS in TCGA, CGGA693, CGGA325, CGGA301 and Rembrandt cohorts, respectively.

SUPPLEMENTARY FIGURE S4 Correlation analysis between the prognostic SERS and clinicopathological characteristics in the CGGA693 cohort. **p* < 0.05, ***p* < 0.01, ****p* < 0.001, and ns No significance.

SUPPLEMENTARY FIGURE S5 Correlation analysis between the prognostic SERS and clinicopathological characteristics in the CGGA325 cohort. **p* < 0.05, ***p* < 0.01, ****p* < 0.001, and ns No significance.

SUPPLEMENTARY FIGURE S6 Correlation analysis between the prognostic SERS and clinicopathological characteristics in the CGGA301 cohort. **p* < 0.05, ***p* < 0.01, ****p* < 0.001, and ns No significance.

SUPPLEMENTARY FIGURE S7 Correlation analysis between the prognostic SERS and clinicopathological characteristics in the Rembrandt cohort. **p* < 0.05, ***p* < 0.01, ****p* < 0.001, and ns No significance.

SUPPLEMENTARY FIGURE S8 The univariate Cox regression and multivariate Cox regression were performed on SERS and other clinicopathological features CGGA693, CGGA325, CGGA301, Rembrandt cohorts, respectively.

SUPPLEMENTARY FIGURE S9 The ROC curves of the nomogram predicted 1-, 3-, and 5-year OS and corresponding calibration curves in CGGA693, CGGA325, CGGA301, Rembrandt cohorts, respectively.

SUPPLEMENTARY FIGURE S10 The distribution of TMB, mutation counts, copy number burdens at focal and arm levels, immune scores, stromal scores, ESTIMATE scores and tumor purity, TIDE, T-cell exclusion score, MSI score and T-cell dysfunction score between high and low-risk groups in TCGA cohort. **P* < 0.05, ***P* < 0.01, and ****P* < 0.001.
