# Supplementary figures and images for: The integrative analysis based on super-enhancer related genes for predicting different subtypes and prognosis of patient with lower-grade glioma
Source: Front Genet. 2023 Apr 7;14:1085584. doi: 10.3389/fgene.2023.1085584 (PMC10119407; doi:10.3389/fgene.2023.1085584)

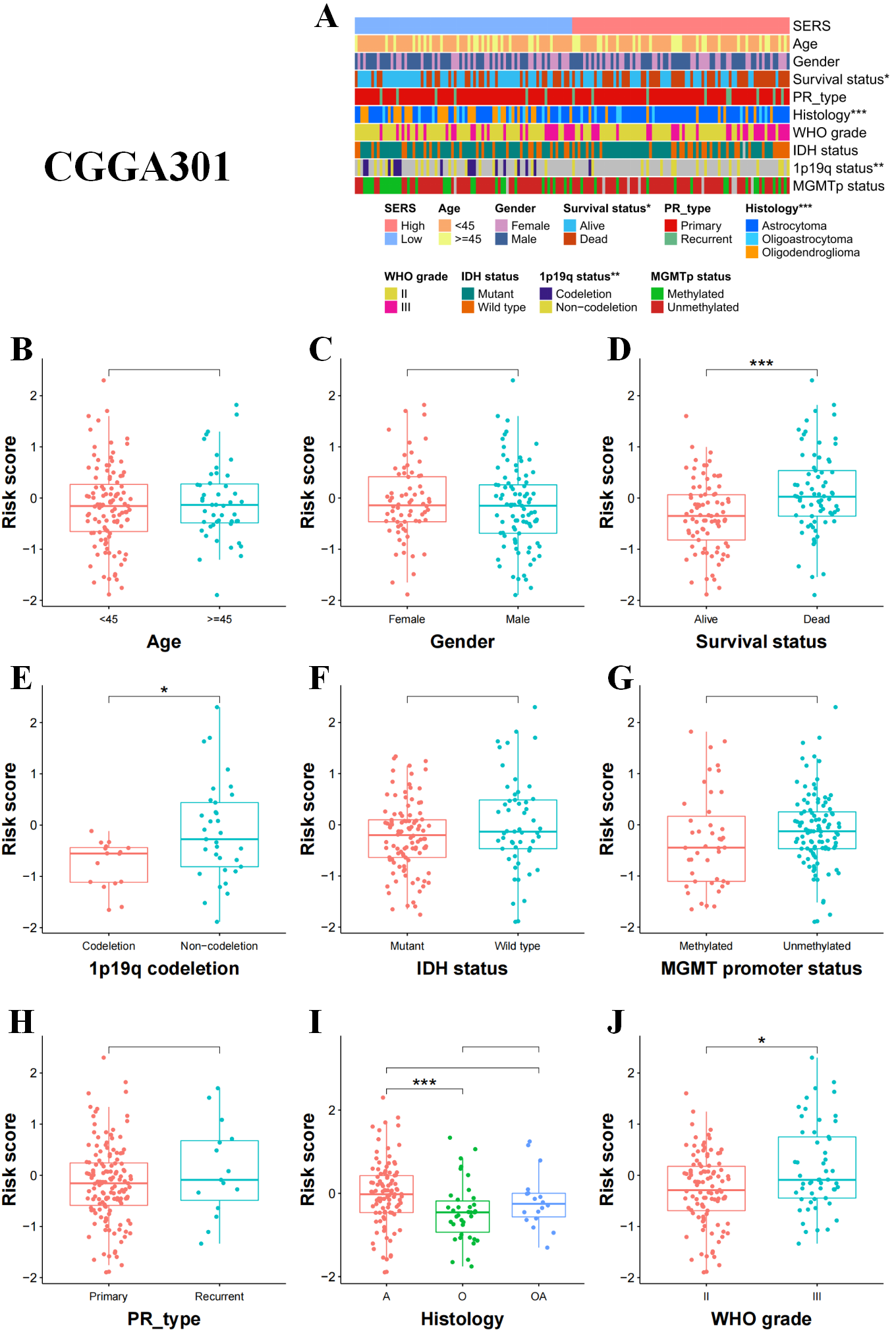

Supplement: Supplementary file 2 [file Image6.TIF]

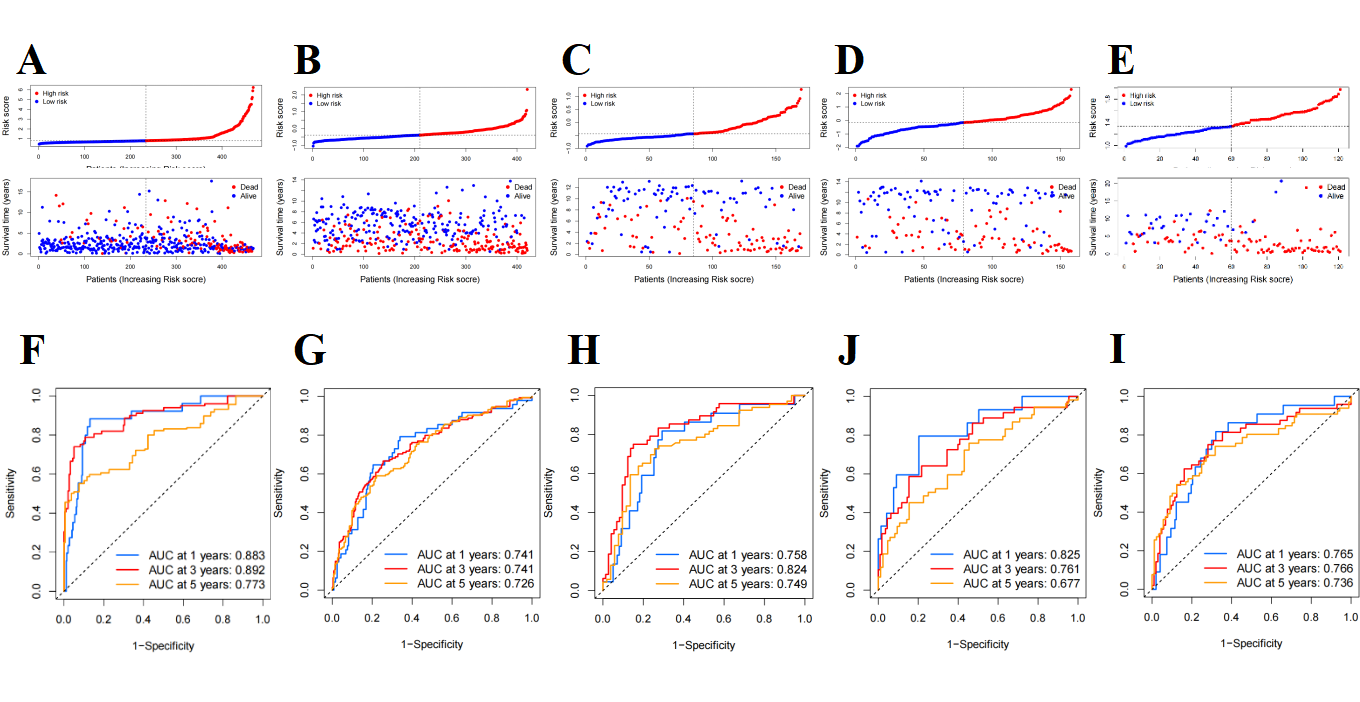

Supplement: Supplementary file 3 [file Image3.TIF]

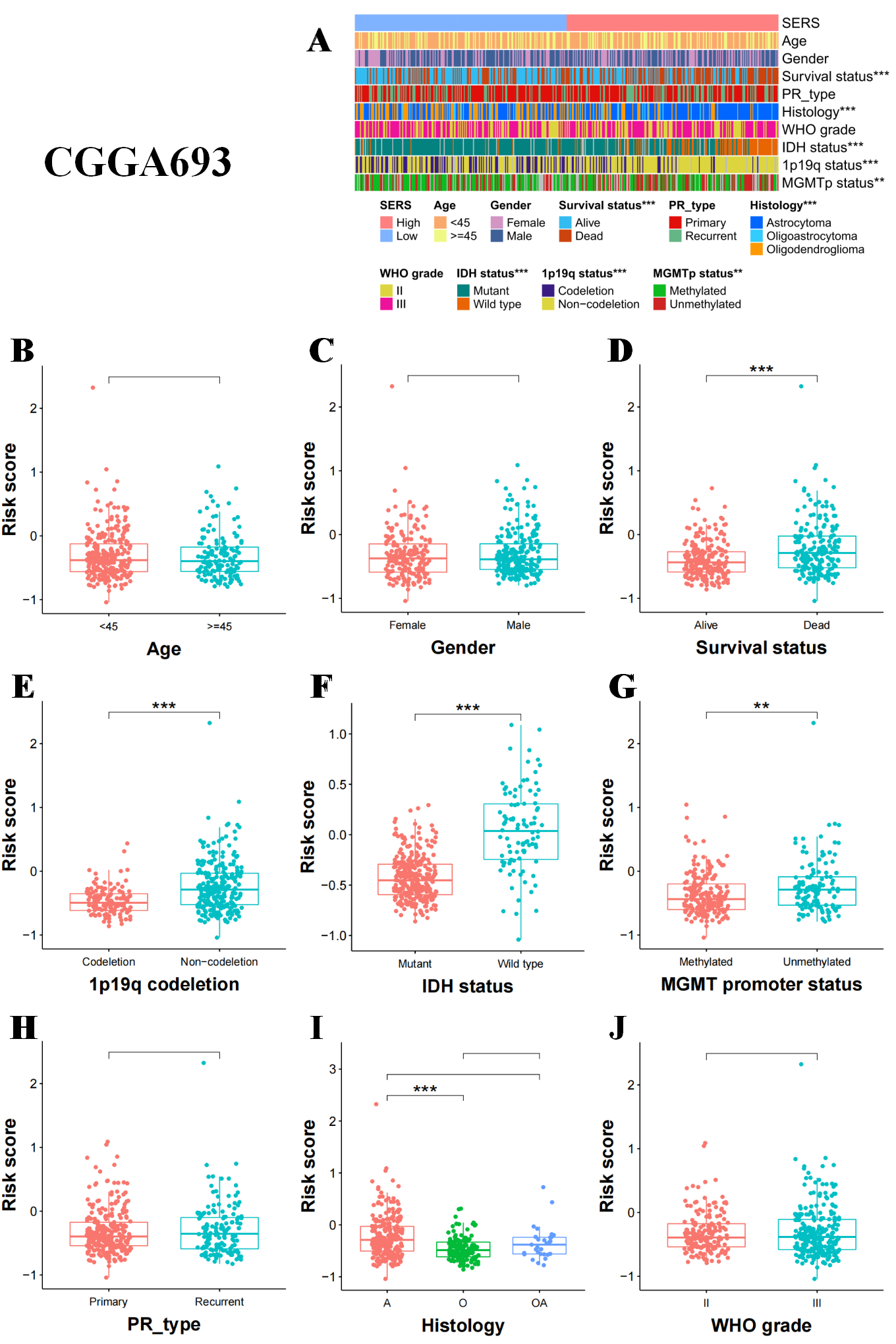

Supplement: Supplementary file 4 [file Image4.TIF]

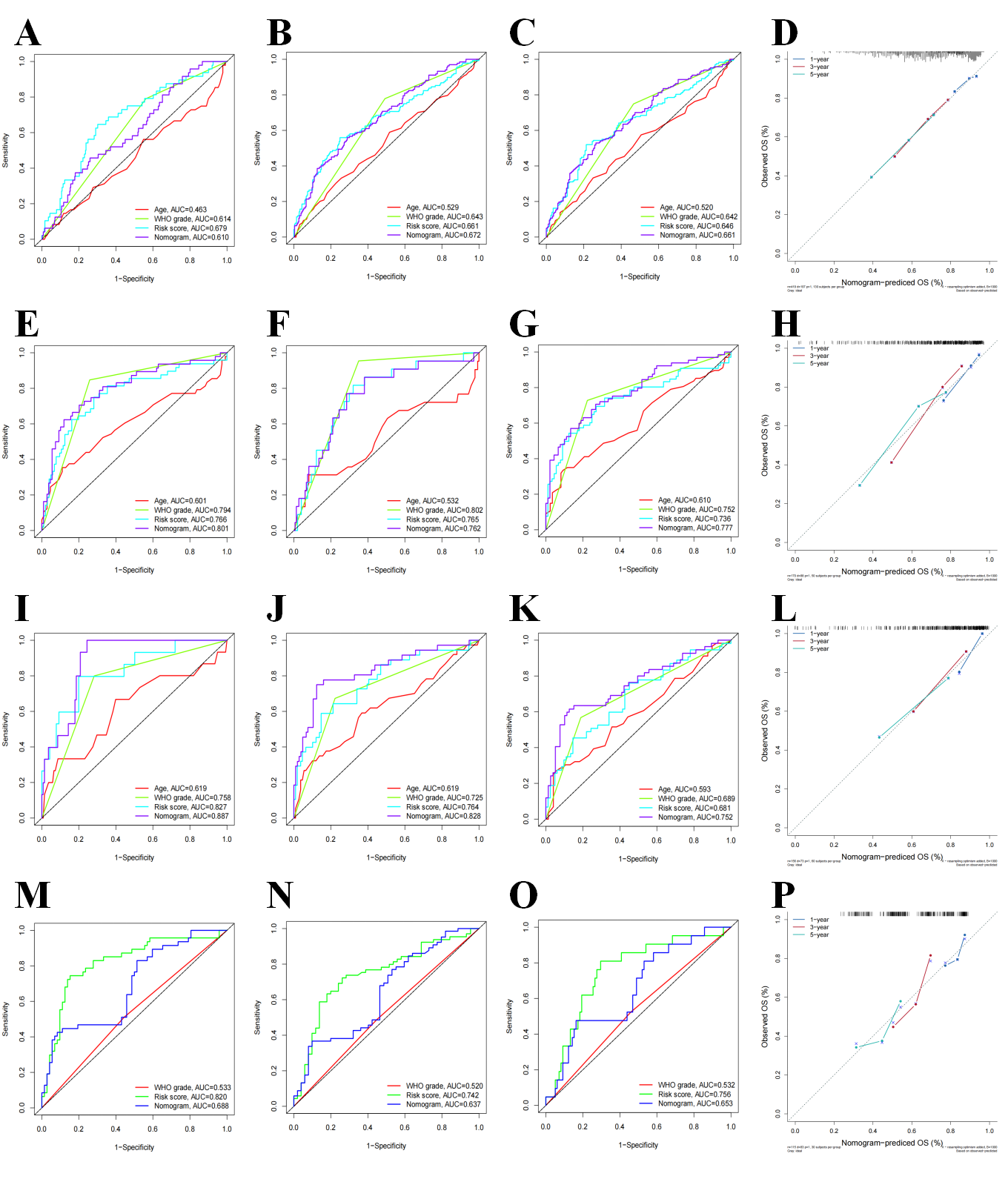

Supplement: Supplementary file 5 [file Image9.TIF]

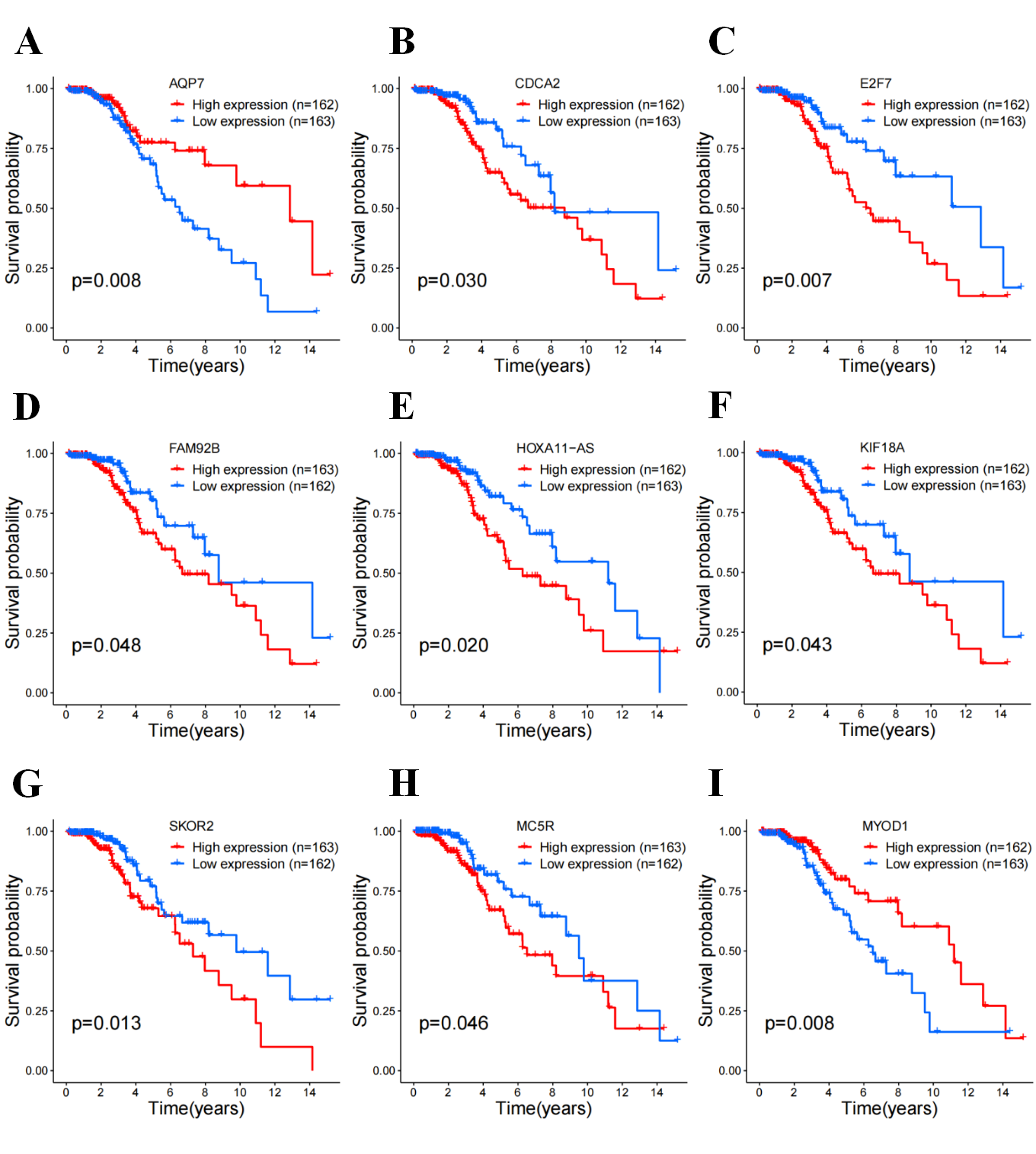

Supplement: Supplementary file 6 [file Image2.TIF]

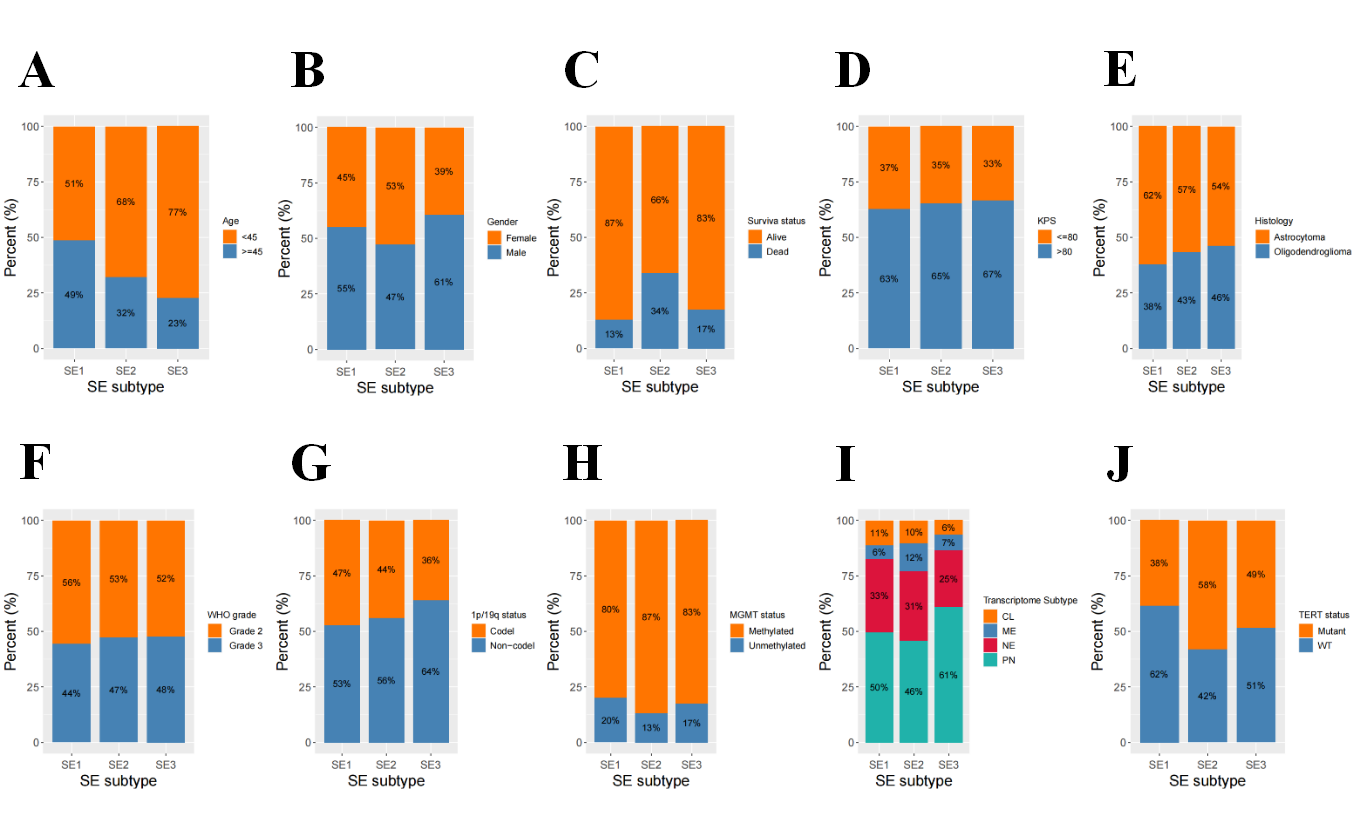

Supplement: Supplementary file 7 [file Image1.TIF]

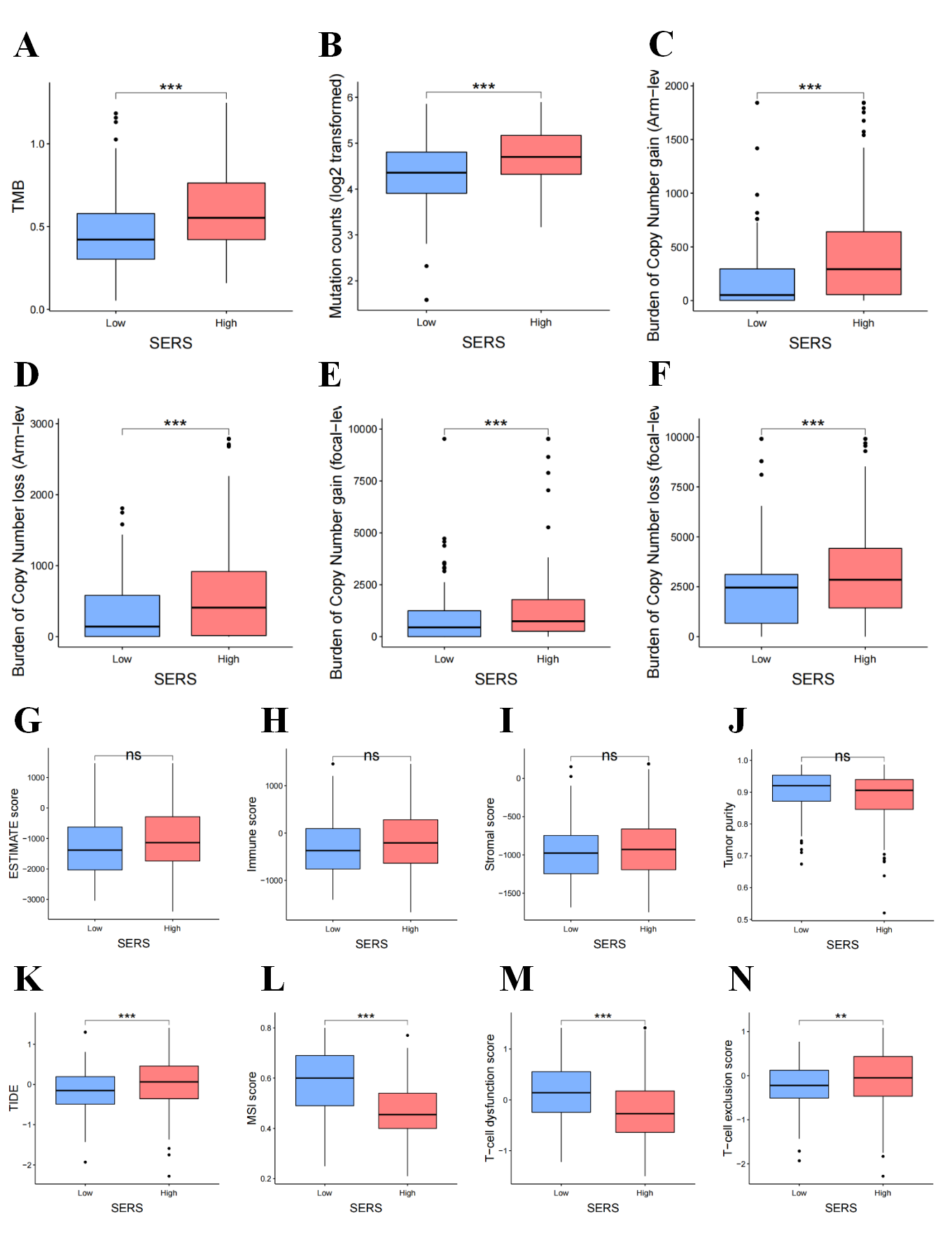

Supplement: Supplementary file 8 [file Image10.TIF]

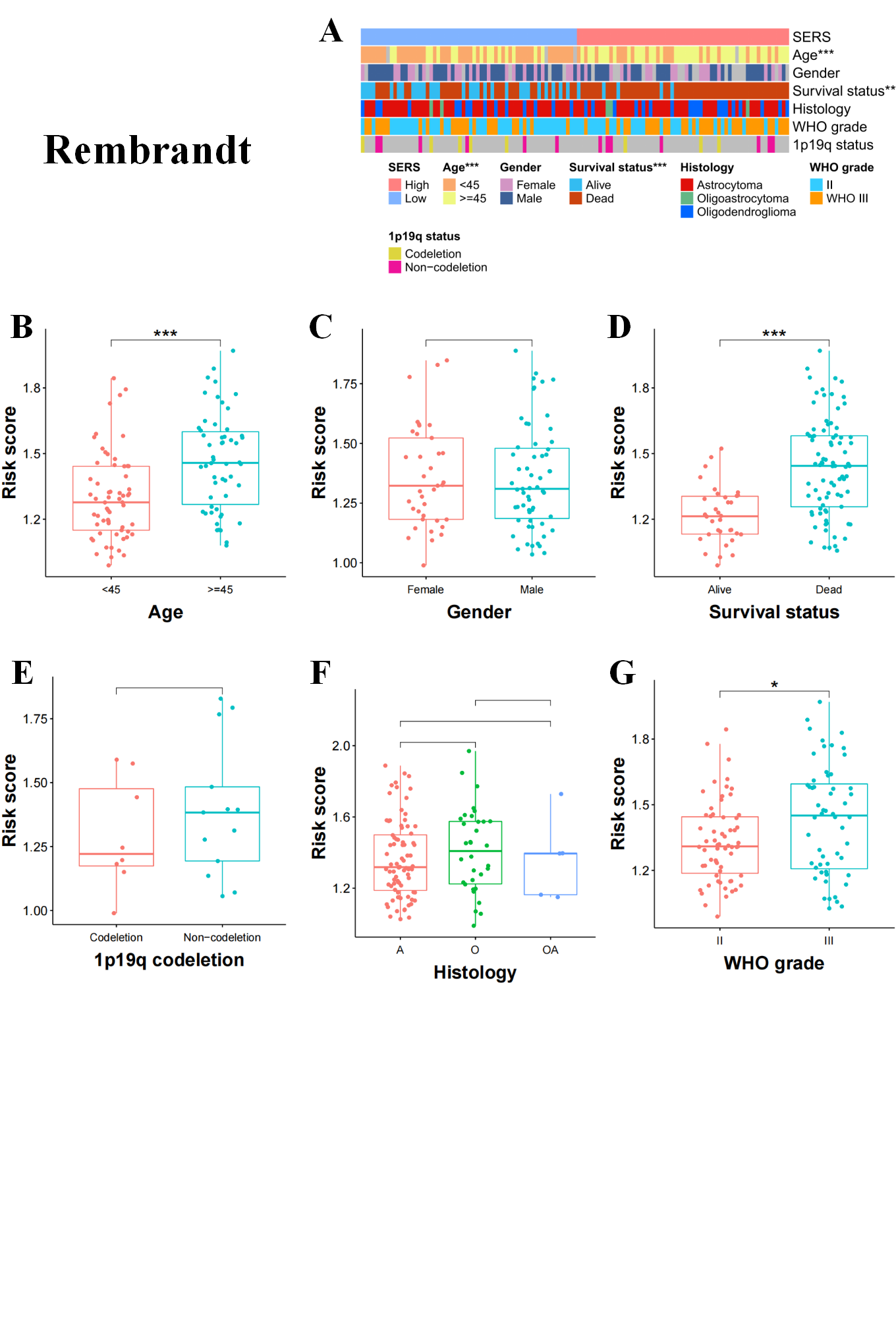

Supplement: Supplementary file 9 [file Image7.TIF]

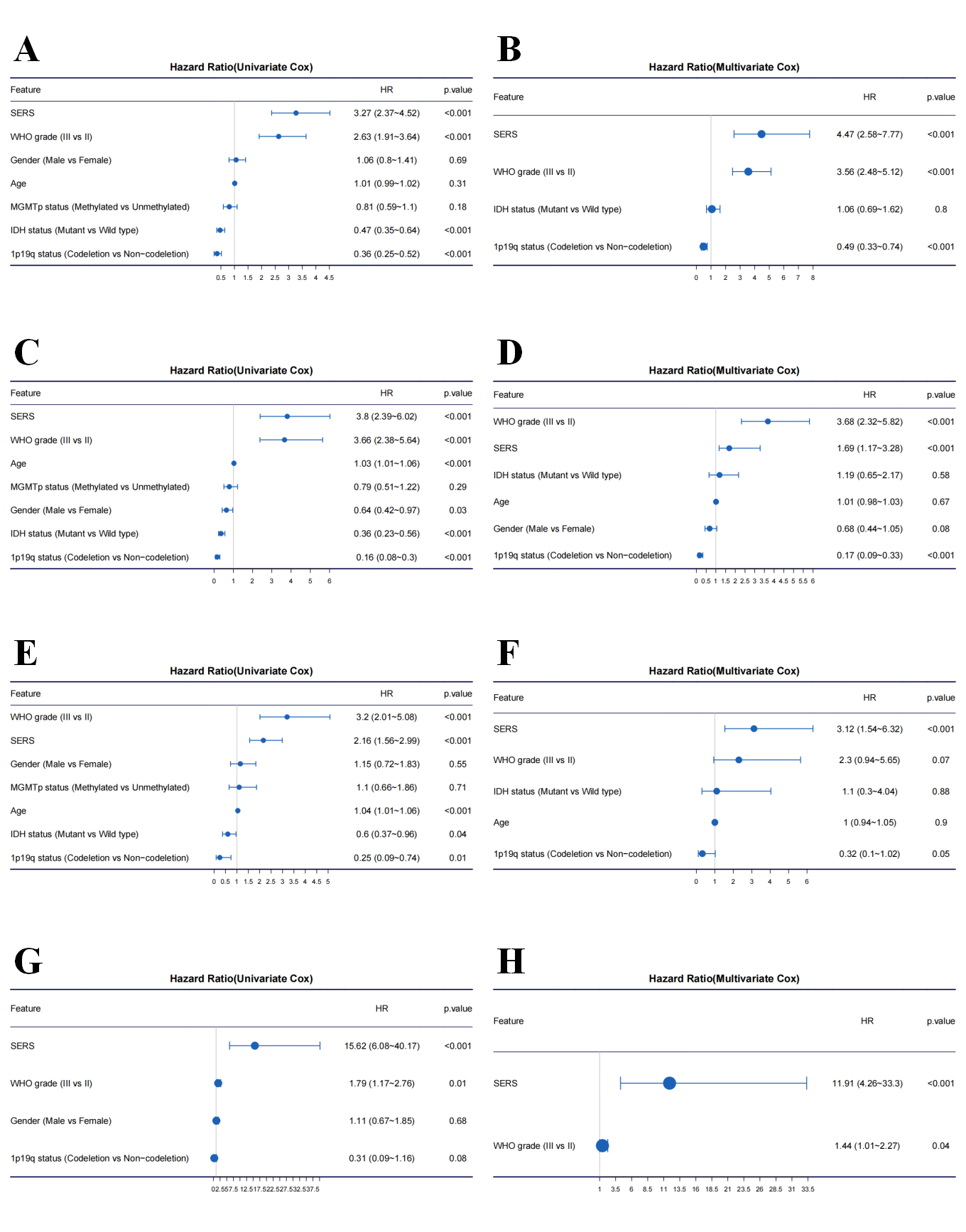

Supplement: Supplementary file 11 [file Image8.TIF]

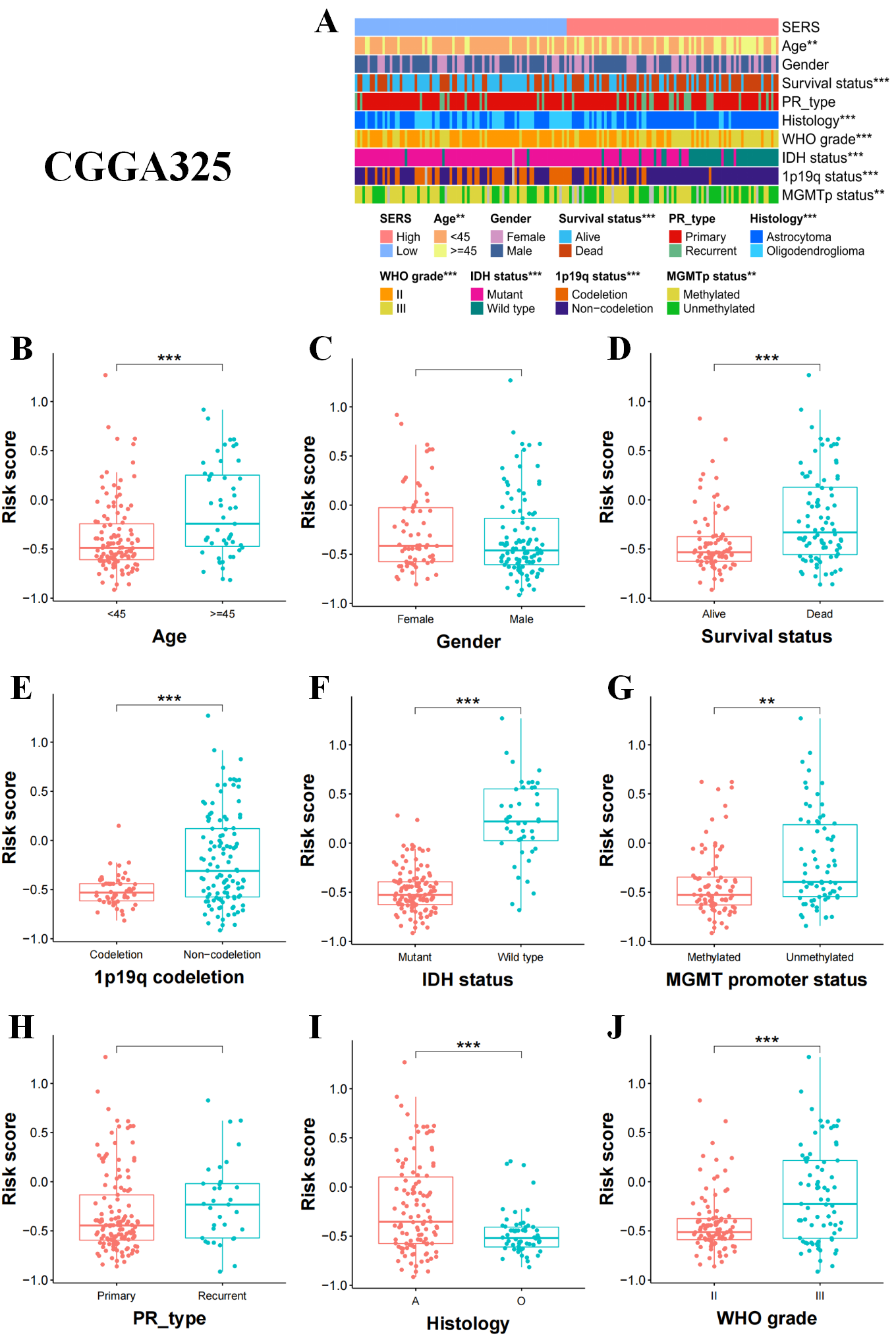

Supplement: Supplementary file 12 [file Image5.TIF]
